# CMPK2 promotes NLRP3 inflammasome activation via mtDNA‐STING pathway in house dust mite‐induced allergic rhinitis

**DOI:** 10.1002/ctm2.70180

**Published:** 2025-01-12

**Authors:** YaoMing Zheng, YaDong Xie, JiaYing Li, YuJie Cao, Min Li, Qing Cao, MiaoMiao Han, HongFei Lou, YiLai Shu, Hui Xiao, HuaBin Li

**Affiliations:** ^1^ Allergy Center, Department of Otolaryngology, Affiliated Eye and ENT Hospital Fudan University Shanghai China; ^2^ State Key Laboratory of Cell Biology, Shanghai Institute of Biochemistry and Cell Biology, Center for Excellence in Molecular Cell Science, Chinese Academy of Sciences University of Chinese Academy of Sciences Shanghai China; ^3^ Department of Otolaryngology, The First Affiliated Hospital College of Medicine Zhejiang University Hangzhou China; ^4^ Ear Center, Department of Otolaryngology, Affiliated Eye and ENT Hospital Fudan University Shanghai China; ^5^ Key Laboratory of Immune Response and Immunotherapy, Shanghai Institute of Immunity and Infection, University of Chinese Academy of Sciences Chinese Academy of Sciences Shanghai China

**Keywords:** allergic rhinitis, CMPK2, mitochondrial DNA, NLRP3 inflammasome, STING

## Abstract

**Background:**

House dust mite (HDM) is the leading allergen for allergic rhinitis (AR). Although allergic sensitisation by inhaled allergens renders susceptible individuals prone to developing AR, the molecular mechanisms driving this process remain incompletely elucidated.

**Objective:**

This study aimed to elucidate the molecular mechanisms underlying HDM‐induced AR.

**Methods:**

We examined the expression of cytidine/uridine monophosphate kinase 2 (CMPK2), STING and the NLRP3 inflammasome in both AR patients and mice. Additionally, we investigated the role of CMPK2 and STING in the activation of the NLRP3 inflammasome in AR.

**Results:**

The expression of CMPK2, STING and the NLRP3 inflammasome was significantly increased in the nasal mucosa of AR patients compared to non‐AR controls. A positive correlation was found between CMPK2 expression and the levels of STING, NLRP3, ASC, CASP1 and IL‐1β. HDM treatment up‐regulated the expression of CMPK2, and CMPK2 overexpression enhanced NLRP3 inflammasome activation in human nasal epithelial cells (HNEPCs). Additionally, mitochondrial reactive oxygen species (mtROS) production following HDM exposure contributed to mitochondrial dysfunction and the release of mitochondrial DNA (mtDNA), which activated the cyclic GMP‐AMP synthase (cGAS)‐STING pathway. Remarkably, depletion of mtDNA or inhibition of STING signalling reduced HDM‐induced NLRP3 inflammasome activation in HNEPCs. In vivo, genetic knockout of CMPK2 or STING alleviated NLRP3 inflammasome activation and ameliorated clinical symptoms of AR in mice.

**Conclusions:**

Our results suggest that HDM promotes the activation of NLRP3 inflammasome through the up‐regulation of CMPK2 and ensuing mtDNA‐STING signalling pathway, hence revealing additional therapeutic target for AR.

**Key points:**

- Cytidine/uridine monophosphate kinase 2 (CMPK2) expression is up‐regulated in the nasal mucosa of patients and mice with allergic rhinitis (AR).
- CMPK2 caused NLRP3 inflammasome activation via mitochondrial DNA (mtDNA)‐STING pathway.
- Blocking CMPK2 or STING signalling significantly reduced the activation of NLRP3 in house dust mite (HDM)‐challenged mice and human nasal epithelial cells (HNEPCs).

## Graphical abstract

1. Cytidine/uridine monophosphate kinase 2 (CMPK2) expression is up‐regulated in the nasal mucosa of patients and mice with allergic rhinitis (AR).
2. CMPK2 caused NLRP3 inflammasome activation via mitochondrial DNA (mtDNA)‐STING pathway.
3. Blocking CMPK2 or STING signalling significantly reduced the activation of NLRP3 in house dust mite (HDM)‐challenged mice and human nasal epithelial cells (HNEPCs).

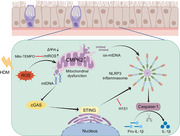

## 1 INTRODUCTION

Allergic rhinitis (AR) results from immunoglobulin E (IgE)‐mediated mucosal inflammation that involves type 2 helper T (Th2) cells affects approximately 5%–50% of the general population with a dramatically increased prevalence.^1^, ^2^, ^3^, ^4^ AR is characterised by nasal pruritus, sneezing, rhinorrhea and nasal congestion, which poses a heavy burden to all patients and society.^5^, ^6^, ^7^ Despite being the most prevalent allergen responsible for respiratory tract allergies, the underlying pathogenic mechanisms of house dust mites (HDMs) in allergic individuals remain inadequately understood.^8^, ^9^, ^10^, ^11^ Given the ubiquity of HDM, it is imperative to accurately understand the pathogenesis of HDM in AR.^12^

As a result of chemical and physical damage triggers, the NLRP3 inflammasome becomes activated, releasing pro‐inflammatory cytokines IL‐1β and IL‐18 in caspase‐1‐dependent fashion.^13^, ^14^, ^15^, ^16^ Recent studies have focused on the NLRP3 inflammasome in the context of allergic diseases.^17^ Furthermore, research shows that NLRP3 activation significantly contributes to the development and severity of AR.^17^, ^18^, ^19^ More importantly, suppression of NLRP3 inflammasome activation could attenuate the inflammatory response and pyroptosis in AR mice, subsequently improve prognosis.^20^, ^21^ Despite the fact that HDM has been shown to activate NLRP3 inflammasomes, the precise molecular regulatory mechanisms remain unclear.^22^, ^23^

Mitochondria are essential not only for energy metabolism but are also considered to be important regulators of innate immunity. Emerging evidence indicates that mitochondrial dysfunction is implicated in the pathogenesis of AR.^24^, ^25^ In this study, we investigated the molecular mechanism of HDM‐induced AR in allergic individuals by analysing the differences of HDM‐induced immune responses between allergic and non‐allergic individuals. The findings revealed that cytidine/uridine monophosphate kinase 2 (CMPK2), a key enzyme in mitochondrial DNA (mtDNA) synthesis, is significantly up‐regulated in HDM‐stimulated allergic individuals. Furthermore, research indicates that CMPK2 facilitates inflammatory responses by enhancing the cytoplasmic release of mtDNA.^26^, ^27^ Multiple pattern recognition receptors (PRRs), including cyclic GMP‐AMP synthase (cGAS), recognise excessively accumulated cytosolic DNA as a danger‐associated molecular pattern (DAMP).^28^, ^29^, ^30^

cGAS is a DNA sensor that activates STING through production of cGAMP, thereby inducing the expression of inflammatory cytokines.^31^, ^32^, ^33^ Notably, recent studies have confirmed that NLRP3 inflammasome activation mediated by the cGAS‐STING pathway is involved in the progression of multiple diseases, such as atherosclerosis,^34^ lung injury,^35^ and liver fibrosis.^36^ However, the role of the cGAS‐STING pathway in the activation of the NLRP3 inflammasome in AR remains unclear. Hence, the purpose of this study is to explore how HDM, an important allergen in AR pathogenesis, regulates the activation of the NLRP3 inflammasome and its molecular mechanisms.

## 2 METHODS

A comprehensive description of the methods employed in this study can be found in the Online Repository of this article

### 2.1 Patients

A total of 30 control subjects and 25 patients with AR were included in this study. More information is provided in this article's Supporting Information.

### 2.2 Microarray data acquisition

The GSE9150 dataset was downloaded from the Gene Expression Omnibus (GEO) database using the GEOquery package in R software.^37^ More details can be found in the Supporting Information.

### 2.3 Data normalisation and identification of DEGs

With the help of the R software package limma, differentially expressed genes (DEGs) in the GSE9150 microarray were identified (adj. *p* < .05, |logFC| > 2).^38^

### 2.4 PPI network construction and hub gene exploration

The protein–protein interaction (PPI) network was built using DEGs via STRING online tool^39^ and visualised with Cytoscape software.^40^ Moreover, Hub genes were identified using the maximal clique centrality (MCC) algorithm in the CytoHubba plugin.^41^

### 2.5 Identification of tissue/organ‐specific expressed genes

An analysis of the tissue distribution of DEGs using BioGPS was conducted to identify tissue‐ or organ‐specific DEG expression.^42^

### 2.6 Cell culture

Human nasal epithelial cells (HNEPCs) were cultured in minimal essential media (MEM) supplemented with 10% foetal bovine serum (FBS) and 1% penicillin/streptomycin.^43^ In some experiments, cells were pre‐incubated with Mito‐TEMPO (Selleck) for 3 h. The culture medium was replaced every 48 h.

### 2.7 H&E staining

Paraffin‐embedded specimens were cut into 3 µm sections and stained with haematoxylin and eosin (H&E). More detailed protocol can be found in the Online Repository.

### 2.8 Immunohistochemical staining

Immunohistochemical (IHC) was conducted as previously described.^44^

### 2.9 Western blotting

We followed previously established protocols for the Western blot analysis, and the antibodies utilised in this study are detailed in Table S2.^44^, ^45^

### 2.10 Immunofluorescence staining

Immunofluorescence (IF) staining for cultured HNEPCs was performed as previously described.^45^

### 2.11 Quantitative RT‐PCR

Quantitative polymerase chain reaction (qPCR) was performed as stated elsewhere.^44^, ^45^

### 2.12 ELISA

A commercial ELISA kit (Ruixin Biotechnology) was used to measure serum levels of total IgE and HDM‐specific IgE in mice.

### 2.13 Transfection

HNEPCs were transfected with small interfering RNA (siRNA) targeting CMPK2 (si‐CMPK2), siRNA targeting STING (si‐STING), cytoplasmic mtDNA or CMPK2 expression plasmids as stated elsewhere.^45^ More details can be found in the Supporting Information.

### 2.14 Extraction and detection of mtDNA

The mtDNA from the cytoplasm of HNEPCs was isolated utilising the Mitochondrial DNA Extraction Kit (ab65321, Abcam) in accordance with the protocol provided by the manufacturer.^46^

### 2.15 Mitochondrial membrane potential (ΔΨm)

After treatment with HDM or Mito‐TEMPO, mitochondrial membrane potential (MMP) of HNEPCs was measured using JC‐1 probe (#C2003S, Beyotime).

### 2.16 Assessment of reactive oxygen species generation

The HNEPCs were incubated for 30 min at 37°C with DCFH‐DA (10 µM, Solarbio) to detect intracellular reactive oxygen species (ROS). For mitochondrial ROS (mtROS) detection, HNEPCs were incubated with MitoSOX (5 µM, YEASEN Biotech) for 10 min at 37°C. After washing, fluorescence images were obtained by a confocal laser‐scanning microscopy (Olympus).

### 2.17 In vivo experiments

AR was induced in mice using HDM (Greer Laboratories). The experimental protocol is depicted in Figure 4A and more information is provided in the Online Repository.

### 2.18 Statistical analyses

All data are presented as means and standard error from the indicated number of samples. Comparative analyses between two groups were conducted utilising a *t*‐test, while comparisons among multiple groups were executed using a one‐way analysis of variance (ANOVA) followed by Bonferroni's test. Non‐normally distributed data were analysed with the Kruskal–Wallis test, and correlations were assessed using the Spearman test. All analyses were conducted with IBM SPSS 22.0, and figures were created using GraphPad Prism 7. A *p* value of <.05 was considered significant.

## 3 RESULTS

### 3.1 Identification of the haematologic/immune system‐specific hub genes associated with allergy

**FIGURE 1:**
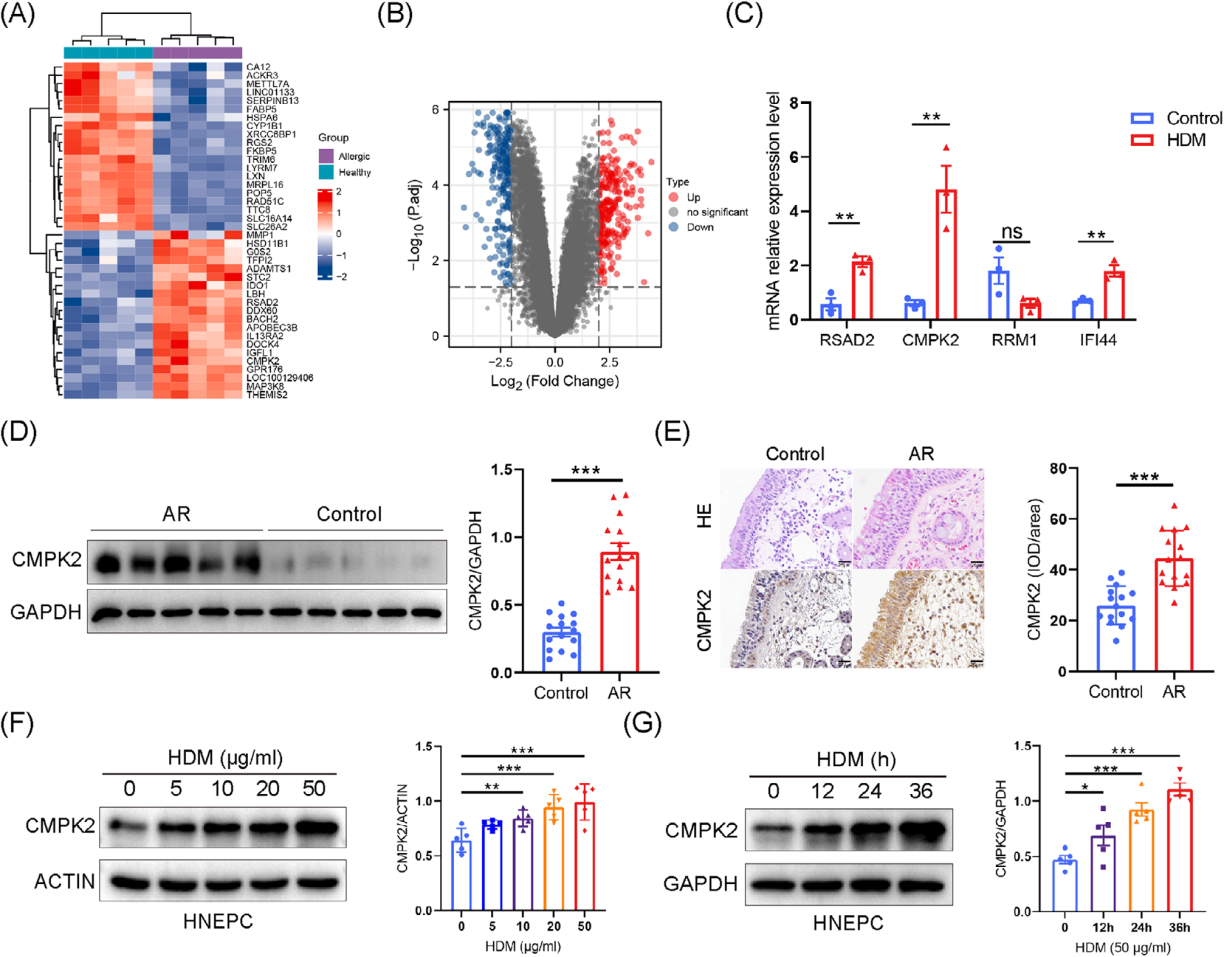
Identification and validation of the haematologic/immune system‐specific expressed hub genes. (A) Heatmap of the top 20 up‐regulated DEGs and the top 20 down‐regulated DEGs (red: high expression; blue: low expression). (B) Volcano plot of DEGs (red: up‐regulated DEGs; blue: down‐regulated DEGs). (C) Expression of mRNA levels of NLRP3, ASC and CASP1 was assessed by quantitative polymerase chain reaction (PCR) in human nasal epithelial cells (HNEPCs) after house dust mite (HDM; 50 µg/mL) treatment for 24 h. (D) The protein levels of cytidine/uridine monophosphate kinase 2 (CMPK2) in nasal mucous tissue in the different study groups was detected by Western blotting. Control, *n* = 15; allergic rhinitis (AR), *n* = 15. (E) Representative photomicrographs and quantitative analysis of CMPK2 in nasal tissue of control subjects (*n* = 15) and patients with AR (*n* = 15; ×400 magnification). (F) HNEPCs were stimulated with HDM at the indicated concentration for 24 h, CMPK2 protein levels were measured by Western blotting (*n* = 5). (G) HNEPCs were stimulated with HDM (50 µg/mL) at the indicated hours. CMPK2 protein levels were measured by Western blotting (*n* = 5). *p < .05, **p < .01 and ***p < .001. DEG, differentially expressed gene; H&E, haematoxylin and eosin; IOD, integral optical density.

First, the datasets GSE9150, which included primary nasal epithelial cells exposed to HDM from five monotypic HDM‐allergic samples and five healthy control samples, were selected for identification of the DEGs between these two groups. Boxplots of the normalised gene expression profiles (Figure S1A), along with principal components analysis (PCA) and Uniform Manifold Approximation and Projection (UMAP), indicated significantly different expression profiles between allergic samples and normal controls (Figure S1B,C). A total of 482 DEGs were identified, with 250 up‐regulated and 232 down‐regulated genes (Figure 1A,B). Among these, 83 are tissue‐/organ‐specific genes as identified by BioGPS (Table S6). The PPI network of the DEGs was constructed utilising the STRING tool and subsequently visualised with Cytoscape (Figure S1D). Subsequently, the top 20 hub genes were determined employing the CytoHubba MCC algorithm, as illustrated in Figure S1D and detailed in Table S7. To investigate the role of HDM pathogenic hub genes, we conducted an intersection analysis between the top 20 hub genes and those specifically expressed in the haematologic/immune system. This analysis led to the identification and validation of four hub genes specific to the haematologic/immune system: RRM1, RSAD2, IFI44 and CMPK2 (Figures 1C and S1F). These tissue‐specific hub genes may play a key role in the pathogenesis of AR, and CMPK2, with the most significant difference, was selected for further study.

### 3.2 Expression of CMPK2 and NLRP3 inflammasome in both nasal mucosa and HNEPCs of AR

Next, Western blotting and immunohistochemistry were used to assess CMPK2 protein levels in nasal tissues from control subjects and AR patients. CMPK2 was detected in nasal epithelial and inflammatory cells in the lamina propria, with its protein levels being significantly higher in individuals with AR than in healthy nasal tissues (Figure 1D,E). Moreover, we explored the expression of CMPK2 in HDM‐stimulated airway epithelial cells. We found that HDM increased CMPK2 protein expression in a dose‐ and time‐dependent manner in HNEPCs (Figure 1F,G). Similar results were obtained using BEAS‐2B cells, which indicates that CMPK2 may be involved in the pathogenesis of AR (Figure S1G,H). After optimisation, HNEPCs were treated with 50 µg/mL HDM allergen for 24 h to establish AR‐HNEPCs for subsequent study.

**FIGURE 2:**
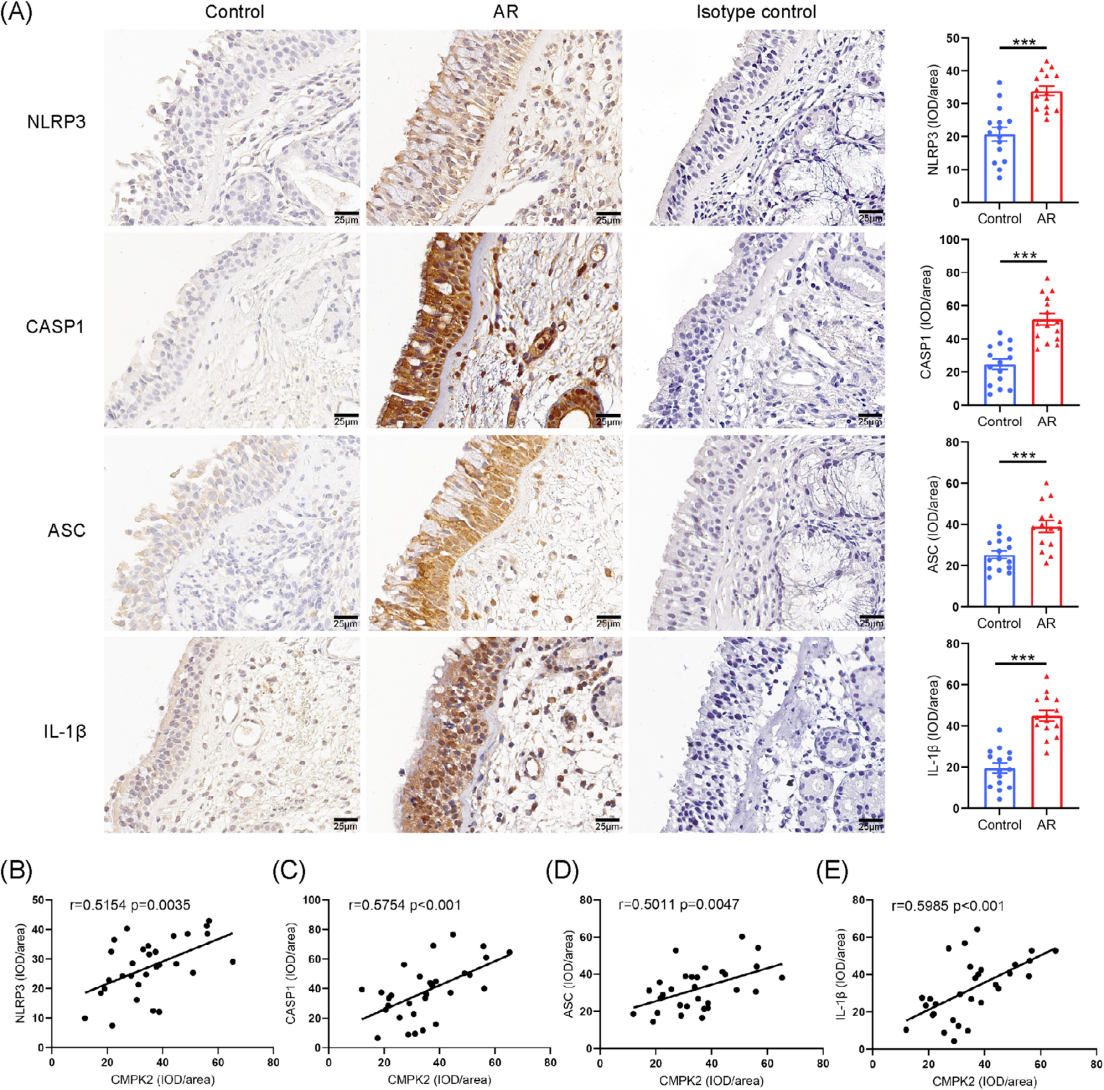
NLRP3 inflammasome‐related proteins increased in patients with allergic rhinitis (AR) and correlated with high expression of cytidine/uridine monophosphate kinase 2 (CMPK2; control, *n* = 15; AR, *n* = 15). (A) Representative photomicrographs and quantitative analysis of NLRP3, ASC, CASP1 and IL‐1β in nasal tissue of control subjects and patients with AR (×400 magnification). (B) Relationship between CMPK2 and NLRP3 in AR. (C) Relationship between CMPK2 and CASP1 in AR. (D) Relationship between CMPK2 and ASC in AR. (E) Relationship between CMPK2 and IL‐1β in AR. *p < .05, **p < .01 and ***p < .001. IOD, integral optical density.

**FIGURE 3:**
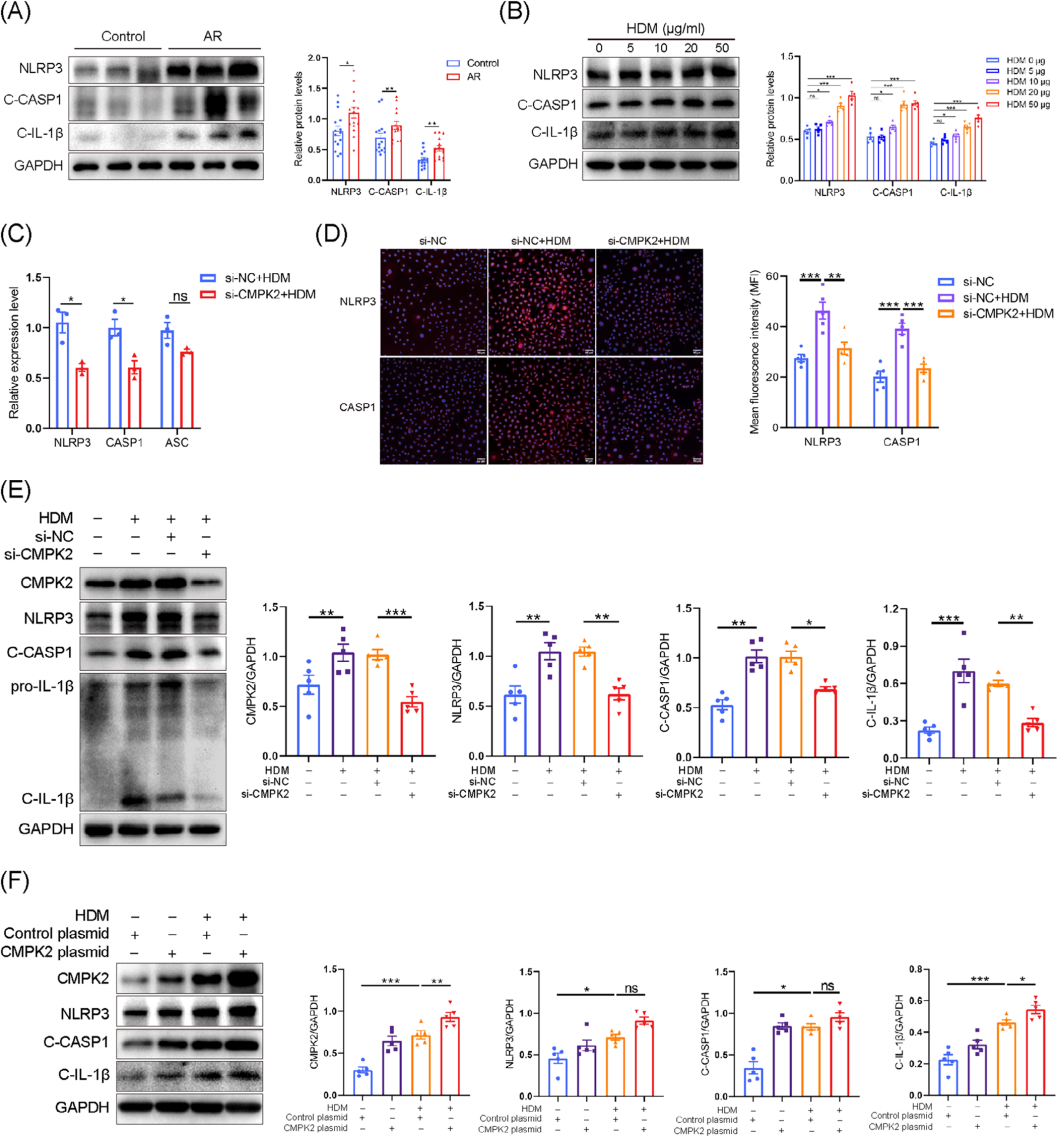
Cytidine/uridine monophosphate kinase 2 (CMPK2) regulates the activation of NLRP3 inflammasome in human nasal epithelial cells (HNEPCs). (A) The protein levels of NLRP3, C‐CASP1 and C‐IL‐1β in nasal mucous tissue in the different study groups were detected by Western blotting. Control, *n* = 15; allergic rhinitis (AR), *n* = 15. (B) HNEPCs were stimulated with house dust mite (HDM) at the indicated concentration for 24 h. The protein levels of NLRP3, C‐CASP1 and C‐IL‐1β were measured by Western blotting (*n* = 5). (C) After si‐CMPK2 transfection, HNEPCs were treated with HDM (50 µg/mL) for 24 h. Cells were collected for reverse transcription polymerase chain reaction (RT‐PCR; *n* = 3). (D) Representative immunofluorescence (IF) staining of NLRP3 and CASP1 (*n* = 5). (E) HNEPCs were transfected with si‐CMPK2 or control small interfering RNA (siRNA) for 24 h and further stimulated with HDM (50 µg/mL) for another 24 h. Cells were collected for Western blotting (*n* = 5). Representative blots are shown, and densitometric analysis of blots was performed. (F) HNEPCs were transfected with CMPK2 plasmid and subsequently stimulated with HDM (50 µg/mL) for 24 h. Cell lysates were harvested for Western blotting. Representative blots are shown, and densitometric analysis of blots was performed (*n* = 5). *p < .05, **p < .01 and ***p < .001. ns = not significant.

In light of previous research indicating that CMPK2 plays an important role in activating NLRP3, we propose that HDMs may enhance NLRP3 activation via CMPK2 up‐regulation, thereby advancing the progression of AR, thus promoting the progression of AR.^47^, ^48^ We initially investigated NLRP3 inflammasome activation in AR patients. IHC staining revealed significantly higher expression of NLRP3, caspase‐1, ASC and IL‐1β in AR nasal tissues compared to controls (Figure 2A). Similar up‐regulation protein trends were observed by Western blot (Figure 3A,B). Notably, correlation analysis revealed that CMPK2 staining intensity was positively correlated with the staining intensities of NLRP3, caspase‐1, ASC and IL‐1β in AR (Figure 2B–E).

### 3.3 CMPK2 is an upstream regulator of the NLRP3 inflammasome

To investigate the potential critical role of CMPK2 in the activation of the NLRP3 inflammasome in AR, we employed siRNA to down‐regulate CMPK2 expression in HNEPCs. The efficacy of the knockdown was subsequently validated through quantitative reverse transcription polymerase chain reaction (qRT‐PCR) and Western blot analysis (Figure S1I,J). Our data showed that knockdown of CMPK2 significantly reduced IL‐1β mRNA expression, but had little effect on the expression of IL‐6, IL‐8, CSF2, periostin and CCL5 (Figure S1K). Moreover, the qRT‐PCR results showed that NLRP3 inflammasome components, including NLRP3 and CASP1, were significantly decreased after CMPK2 knockdown (Figure 3C). Consistently, the NLRP3 inflammasome activation was mitigated by knocking down CMPK2, as shown by reduced cleavage of CASP1 and IL‐1β in response to HDM stimulation (Figures 3E and S2A,B). Similar results were observed by IF staining (Figure 3D). Moreover, we transfected HNEPCs with a CMPK2 expression plasmid (transfection efficiencies shown in Figure S2C), and CMPK2 overexpression significantly increased C‐IL‐1β expression (Figure 3F). This indicates that CMPK2 is essential for NLRP3 inflammasome activation in response to HDM stimuli.

### 3.4 CMPK2 deficiency impairs NLRP3 inflammasome activation in HDM‐challenged AR mice

**FIGURE 4:**
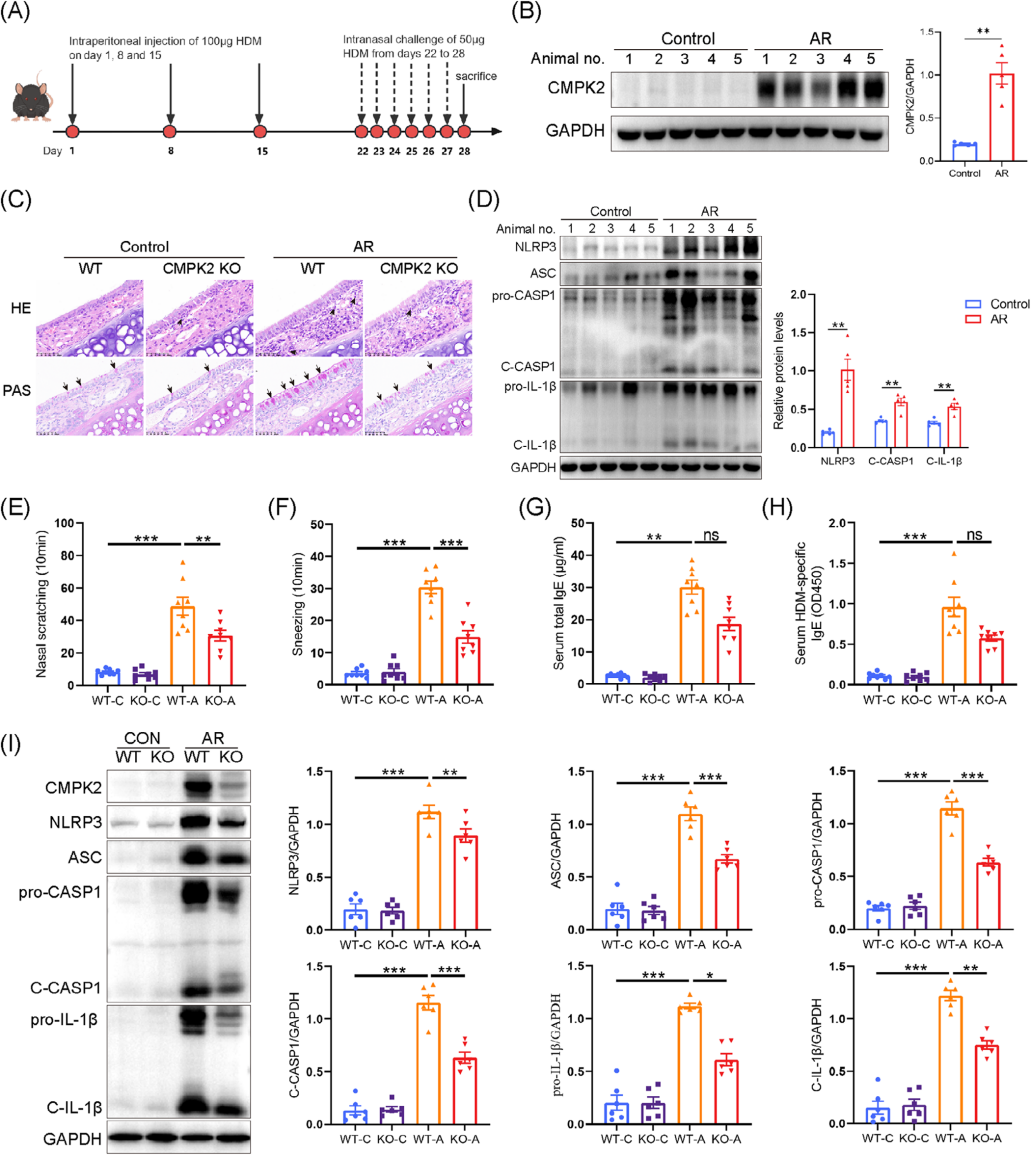
Cytidine/uridine monophosphate kinase 2 (CMPK2) deficiency suppresses the activation of NLRP3 inflammasome in house dust mite (HDM)‐challenged allergic rhinitis (AR) mice. (A) Schematic representation of the experimental protocol. (B) The CMPK2 protein levels in the nasal tissues of mice (*n* = 5) with or without AR were determined by Western blotting. (C) Eosinophil infiltration and goblet cell metaplasia were evaluated in haematoxylin and eosin (H&E)‐ or PAS‐stained sections of wild‐type (WT) and CMPK2^–/–^ mice with and without AR, respectively (×400 magnification). (D) Expression of protein levels of NLRP3 inflammasome in AR mice (*n* = 5). (E, F) Number of nasal scratching (E) and sneezing (F) in AR mice (*n* = 8). (G) Total immunoglobulin E (IgE) levels in serum (*n* = 8). (H) HDM‐specific IgE levels in serum (*n* = 8). (I) Expression of protein levels of CMPK2 and NLRP3 inflammasome in WT and CMPK2^–/–^ mice with and without AR (*n* = 6). *p < .05, **p < .01 and ***p < .001. ns = not significant.

Subsequently, we aimed to investigate whether a deficiency in CMPK2 mitigates the progression of AR in vivo. According to the experimental schedule depicted in Figure 4A, a mice model of AR induced by HDM was established. In alignment with the findings derived from human samples and HNEPCs, the protein expression levels of CMPK2 and the NLRP3 inflammasome‐related proteins (NLRP3, ASC, C‐CASP1, C‐IL‐1β) were significantly elevated in the nasal mucosa of mice with HDM‐induced AR when compared to those of the control group (Figure 4B,D). In order to further elucidate the role of CMPK2 in activating the NLRP3 inflammasome in vivo, we used CMPK2^−/−^ mice. As shown in Figure S3, CMPK2 was not expressed in the nasal mucosa of CMPK2‐deficient mice. The H&E staining demonstrated an increased number of eosinophils in the nasal mucosa of HDM‐challenged wild‐type (WT) mice, whereas a reduced number was observed in CMPK2^−/−^ mice (Figure 4C). Furthermore, the number of periodic acid schiff (PAS)‐stained goblet cells in HDM‐challenged mice was significantly elevated compared to control mice, and CMPK2 knockout attenuated goblet cell hyperplasia (Figure 4C). In addition, HDM‐sensitised mice had markedly increased frequency of sneezing and nose scratching compared with the control group, but CMPK2 knockout relieved these allergic symptoms (Figure 4E,F). Similarly, the elevation in serum total IgE and HDM‐specific IgE levels in WT mice after HDM challenge was ameliorated in CMPK2 knockout mice (Figure 4G,H). Notably, a significant reduction in NLRP3 inflammasome activation occurred in the nasal mucosa of HDM‐challenged AR mice with CMPK2 deficiency (Figure 3I). Furthermore, the IHC staining results exhibited a similar trend of attenuated inflammasome activation in CMPK2‐deficient mice (see Figure S4A). These findings indicate that CMPK2 plays a role in the pathogenesis of AR by facilitating the activation of the NLRP3 inflammasome.

### 3.5 mtROS played a key role in HDM‐induced activation of the NLRP3 inflammasome in HNEPCs

**FIGURE 5:**
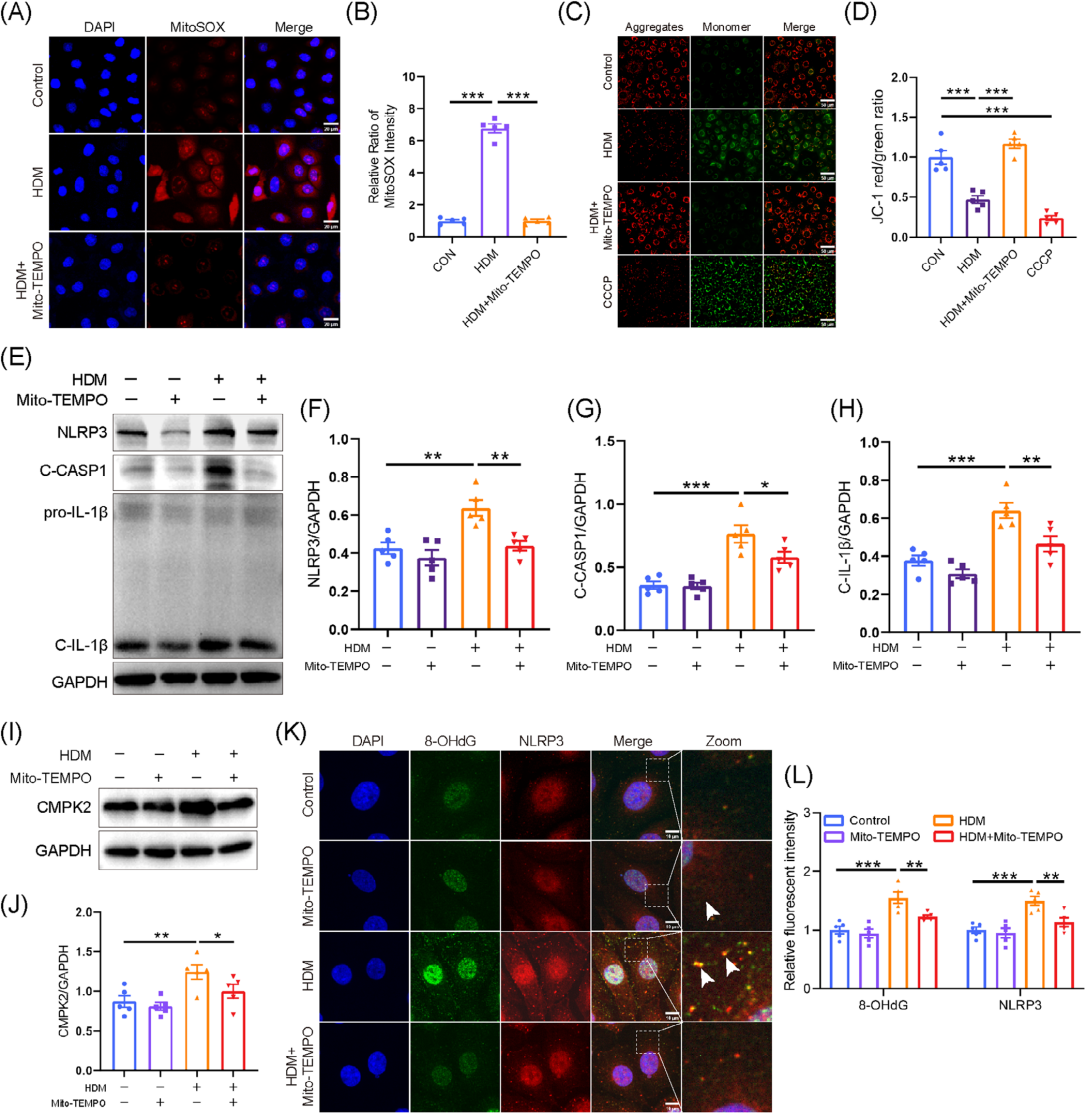
Mito‐TEMPO can inhibit the activation of NLRP3 inflammasome by inhibiting the accumulation of mitochondrial reactive oxygen species (mtROS) in human nasal epithelial cells (HNEPCs). HNEPCs pretreated with Mito‐TEMPO (50 µmol/L) for 3 h and then stimulated with house dust mite (HDM; 50 µg/mL) for 24 h. The HDM group was treated with HDM (50 µg/mL) for 24 h. (A, B) Representative fluorescence images of mitochondrial ROS staining using the MitoSOX probe (*n* = 5). Scale bar = 20 µm. (C, D) Representative fluorescence images of JC‐1 staining to evaluate MMP in HNEPCs (*n* = 5). Scale bar = 50 µm; red, aggregates; green, monomers. (E–H) Representative blots and quantitative analysis of NLRP3, C‐CASP1 and C‐IL‐1β relative protein expression in HNEPCs (*n* = 5). (I, J) Representative blots of cytidine/uridine monophosphate kinase 2 (CMPK2) expression in HNEPCs treated with HDM and Mito‐TEMPO (*n* = 5). (K, L) Expression of immunofluorescence (IF) costaining of 8‐OHdG/NLRP3 in HNEPCs (*n* = 5). Scale bar = 10 µm. *p < .05, **p < .01 and ***p < .001.

We conducted a further investigation into the mechanisms underlying the activation of the NLRP3 inflammasome induced by HDM in HNEPCs. We first investigated the relationship between HDM and mitochondrial dysfunction, which may lead to mtDNA leakage into the cytosol, thereby activating the NLRP3 inflammasome.^49^ After HDM treatment, the green fluorescence representing intracellular ROS increased significantly (Figure S5A). Subsequently, our findings revealed an elevation in mtROS in HDM‐treated HNEPC, as demonstrated by IF staining (Figure S5B). Furthermore, HDM exposure resulted in a decrease in mitochondrial membrane potential, as illustrated by a reduction in JC‐1 aggregates, and an increase in JC‐1 monomers (Figure S5C). To further substantiate the contributory role of mtROS, Mito‐TEMPO, a specific mtROS scavenger, was employed. Our study showed that Mito‐TEMPO (50 µmol/L) pretreatment significantly reduced HDM‐induced mtROS accumulation and restored mitochondrial membrane potential (Figure 5A–D). Furthermore, Western blot analysis revealed that Mito‐TEMPO effectively reversed the up‐regulation of CMPK2 and the activation of the NLRP3 inflammasome in AR (Figure 5E–J).

Previous studies indicate that mtROS can oxidise mitochondrial DNA (ox‐mtDNA), leading to its release into the cytoplasm, where it activates the inflammasome by binding to NLRP3.^50^, ^51^, ^52^ We then used a fluorescent antibody to label 8‐OHdG, a marker of DNA oxidation. Consistent with previous findings, the oxidised DNA was increased in HNEPCs after HDM treatment and co‐localised with cytosolic NLRP3, which can promote the activation of NLRP3 inflammasome (Figure 5K,L). However, pretreatment with Mito‐TEMPO markedly reversed this phenomenon (Figure 5K,L). These data indicated that Mito‐TEMPO treatment dramatically alleviated mitochondrial oxidative stress and mitochondrial dysfunction, which may serve as a potential target for AR therapy.

### 3.6 CMPK2 facilitates NLRP3 inflammasome activation through mtDNA

**FIGURE 6:**
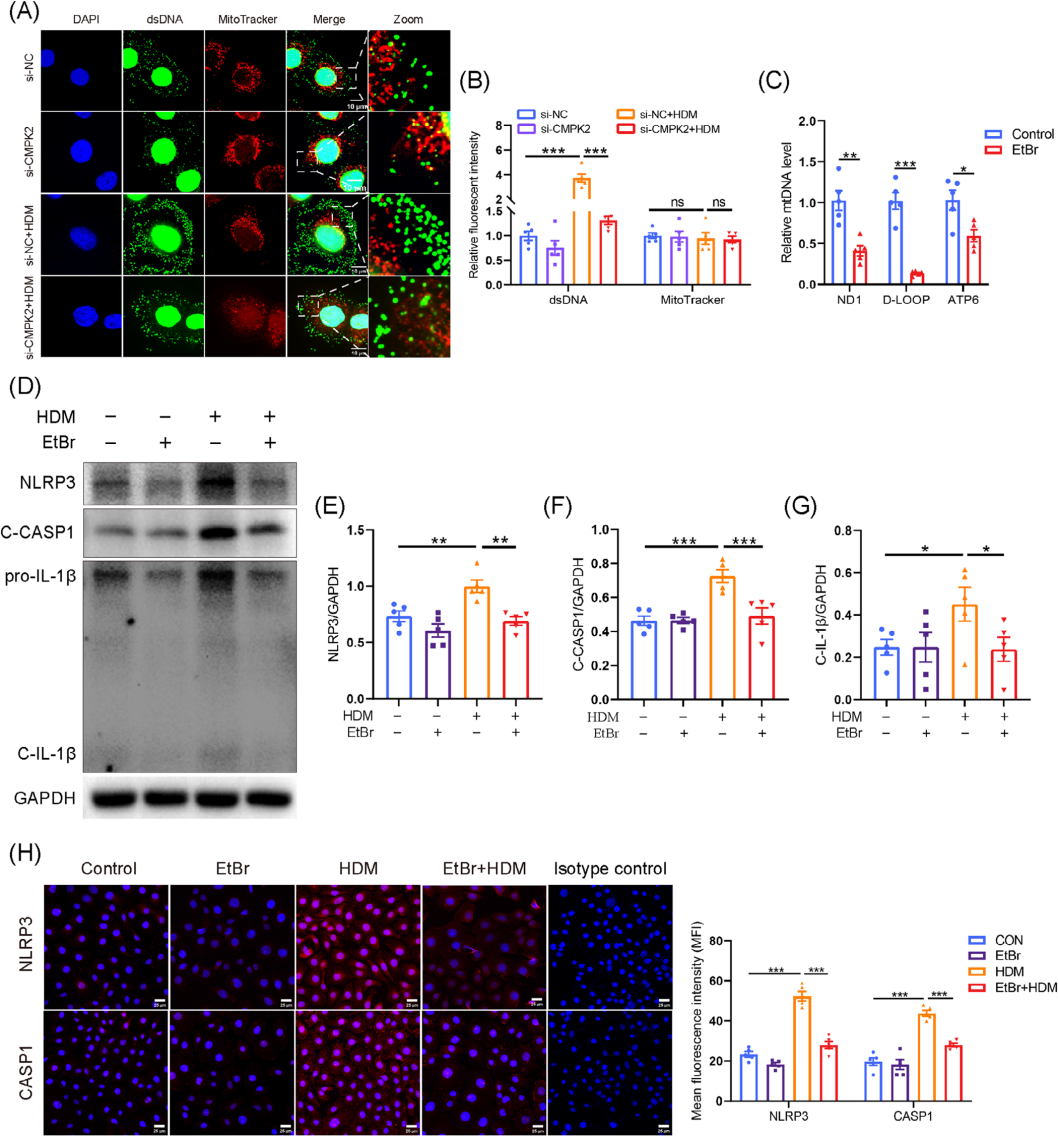
Cytosolic mitochondrial DNA (mtDNA) is necessary for the house dust mite (HDM)‐induced activation of NLRP3 inflammasome in human nasal epithelial cells (HNEPCs). (A, B) HNEPCs were transfected with si‐cytidine/uridine monophosphate kinase 2 (CMPK2) or control small interfering RNA (siRNA) for 24 h and further stimulated with HDM (50 µg/mL) for another 24 h. Representative fluorescence images of dsDNA (green) and mitochondria (red) in HNEPCs. Scale bar = 10 µm. (C) mRNA levels of ND1, D‐LOOP and ATP6 were assessed by quantitative real‐time polymerase chain reaction (qPCR) in HNEPCs after EtBr stimulation (2 µg/mL, 24 h; *n* = 5). (D–G) HNEPCs pretreated with EtBr (2 µg/mL) for 24 h and then stimulated with HDM (50 µg/mL) for another 24 h. The HDM group was treated with HDM (50 µg/mL) for 24 h (*n* = 5). (H) Representative immunofluorescence (IF) staining and quantitative analysis of NLRP3 and CASP1. Scale bar = 25 µm. *p < .05, **p < .01 and ***p < .001.

Recently, it was found that mtDNA is essential for NLRP3 inflammasome activation and that this process is dependent on the mitochondrial nucleotide monophosphate kinase CMPK2, which is a critical enzyme responsible for mtDNA synthesis.^26^ Our findings revealed that stimulation with HDM significantly increased the mtDNA copy number in HNEPCs compared to the control group, whereas knockdown of CMPK2 resulted in a substantial reduction in mtDNA copy number (Figure S6A,B). In addition, our study showed that mtDNA in the cytoplasm of HNEPCs was significantly increased after HDM treatment (Figure S6C–E), and knockdown of CMPK2 significantly attenuated this effect (Figure 6A,B).

To elucidate the role of cytosolic mtDNA in the activation of the NLRP3 inflammasome triggered by HDM, we employed EtBr to inhibit mtDNA replication.^53^ After the application of EtBr, the mtDNA copy number of HNEPCs was reduced by approximately 60% (Figure 6C). As expected, the Western blotting results showed that NLRP3 inflammasome‐related protein (NLRP3, C‐CASP1 and C‐IL‐1β) levels were significantly up‐regulated in HNEPCs treated with HDM compared with the levels in the untreated controls. However, such elevation of the NLRP3 inflammasome was inhibited by EtBr pretreatment (Figure 6D–G). Furthermore, immunofluorescent staining results showed a similar trend to the Western blot assay results (Figure 6H). The findings indicate that mtDNA serves as a crucial mediator in the activation of the NLRP3 inflammasome by CMPK2, potentially involving the cGAS‐STING signalling pathways.

### 3.7 Enhanced cGAS‐STING pathway in HDM‐induced AR

**FIGURE 7:**
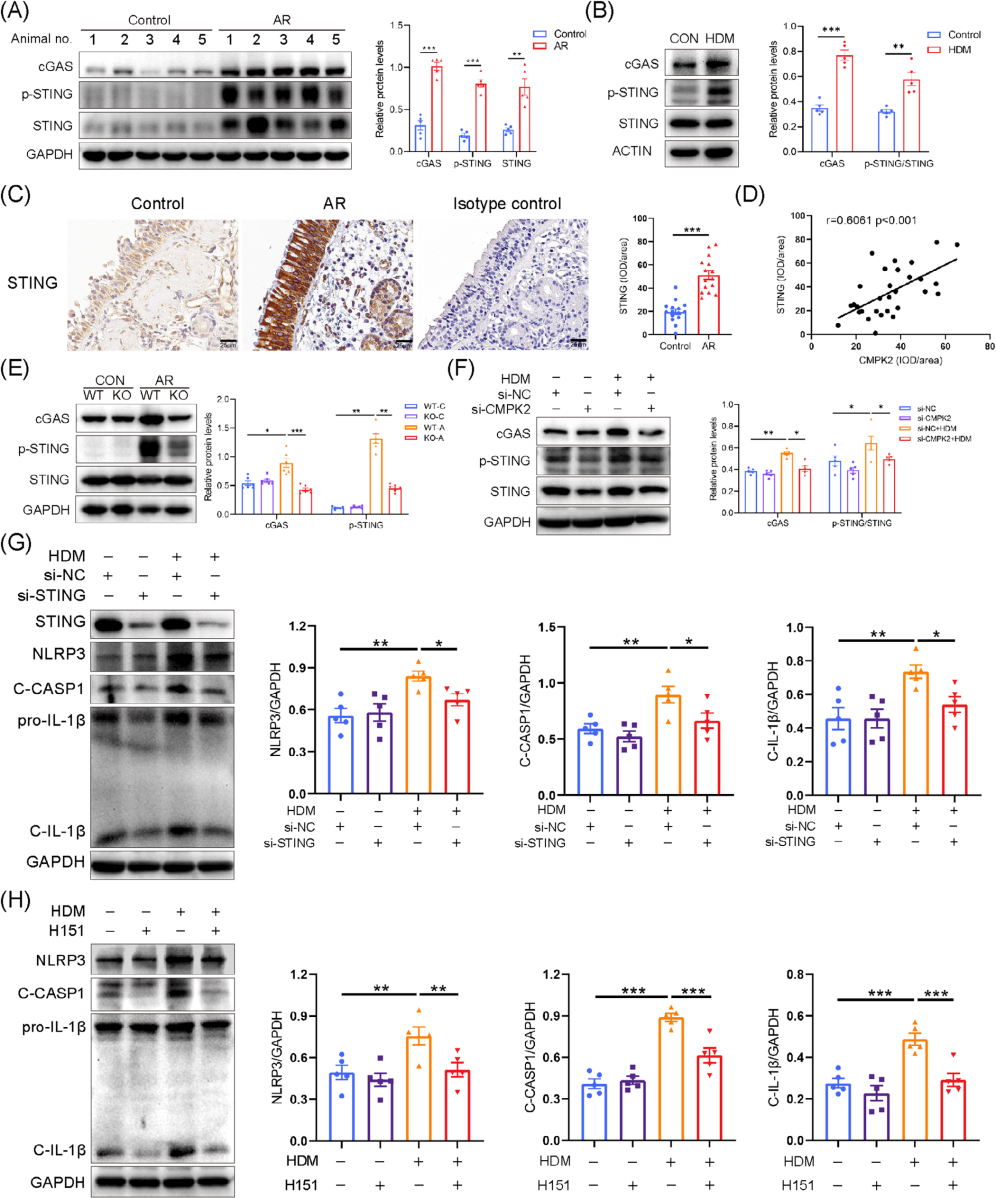
House dust mite (HDM)‐driven NLRP3 inflammasome activation relies on the cyclic GMP‐AMP synthase (cGAS)‐STING pathway. (A) The expression of cGAS, STING and p‐STING in the nasal tissues of mice (*n* = 5) with or without allergic rhinitis (AR) was determined by Western blotting. (B) The expression of cGAS, STING and p‐STING in human nasal epithelial cells (HNEPCs) after stimulation with HDM (50 µg/mL) for 24 h was determined by Western blotting (*n* = 5). (C) Immunohistochemical staining for STING in nasal tissue of control subjects and patients with AR (*n* = 15; ×400 magnification). (D) Correlations between cytidine/uridine monophosphate kinase 2 (CMPK2) and STING in nasal mucosa of AR patients. Spearman test. IOD, integrated optical density. (E) Western blotting for cGAS, STING and p‐STING in the nasal tissues of wild‐type (WT) and CMPK2^–/–^ mice with and without AR, respectively (*n* = 6). (F) After knockdown of CMPK2 in HNEPCs, the effect of HDM (50 µg/mL) on the activation of cGAS‐STING pathway was detected (*n* = 5). (G) After si‐STING transfection, HNEPCs were treated with HDM (50 µg/mL) for 24 h. Representative blots and quantitative analysis of STING, NLRP3, C‐CASP1 and C‐IL‐1β relative protein expression in HNEPCs (*n* = 5). (H) HNEPCs pretreated with H151 (10 µM) for 3 h and then stimulated with HDM (50 µg/mL) for 24 h. The HDM group was treated with HDM (50 µg/mL) for 24 h. Representative blots and quantitative analysis of NLRP3, C‐CASP1 and C‐IL‐1β relative protein expression in HNEPCs (*n* = 5). *p < .05, **p < .01 and ***p < .001.

Studies have indicated that the cGAS‐STING pathway is crucial for activating the NLRP3 inflammasome. Our experiments showed that HDM stimulation increased cGAS protein expression and STING phosphorylation both in vivo (Figure 7A) and in vitro (Figure 7B), indicating activation of the cGAS‐STING pathway in the AR model. Furthermore, the IHC staining results indicated that STING expression was elevated in patients with AR relative to the control group (Figure 7C). Additionally, there was a positive correlation between the intensity of CMPK2 in IHC staining and STING expression in AR patients (Figure 7D). However, the absence of CMPK2 abrogated STING activation in the AR model (Figures 7E,F and S7A). Taken together, these results suggest that the cGAS‐STING pathway is activated in HDM‐induced AR and is regulated by CMPK2.

### 3.8 CMPK2 promotes NLRP3 inflammasome activation via the cGAS/STING pathway

To further explore whether cGAS‐STING participates in the activation of NLRP3 inflammasome in HNEPCs, STING siRNA and H151 (a selective STING antagonist) were used to inhibit cGAS‐STING signalling. The knockdown efficiencies of the siRNAs were validated in HNEPCs by Western blot and qRT‐PCR (Figure S7B,C). We found that si‐STING treatment or pharmacological inhibition of STING with H‐151 inhibited HDM‐induced activation of NLRP3 inflammasome in HNEPCs (Figure 7G,H). To verify whether cytosolic mtDNA activates NLRP3 via the cGAS‐STING pathway, we isolated mtDNA and transfected it into HNEPCs. Western blot analysis demonstrated that the transfection of cytoplasmic mtDNA significantly activated the STING signalling pathway and subsequently induced the activation of the NLRP3 inflammasome. This activation was notably inhibited upon STING knockdown (Figure S8A,B). Furthermore, pretreatment of Mito‐TEMPO can also reduce the phosphorylation level of STING (Figure S9A).

**FIGURE 8:**
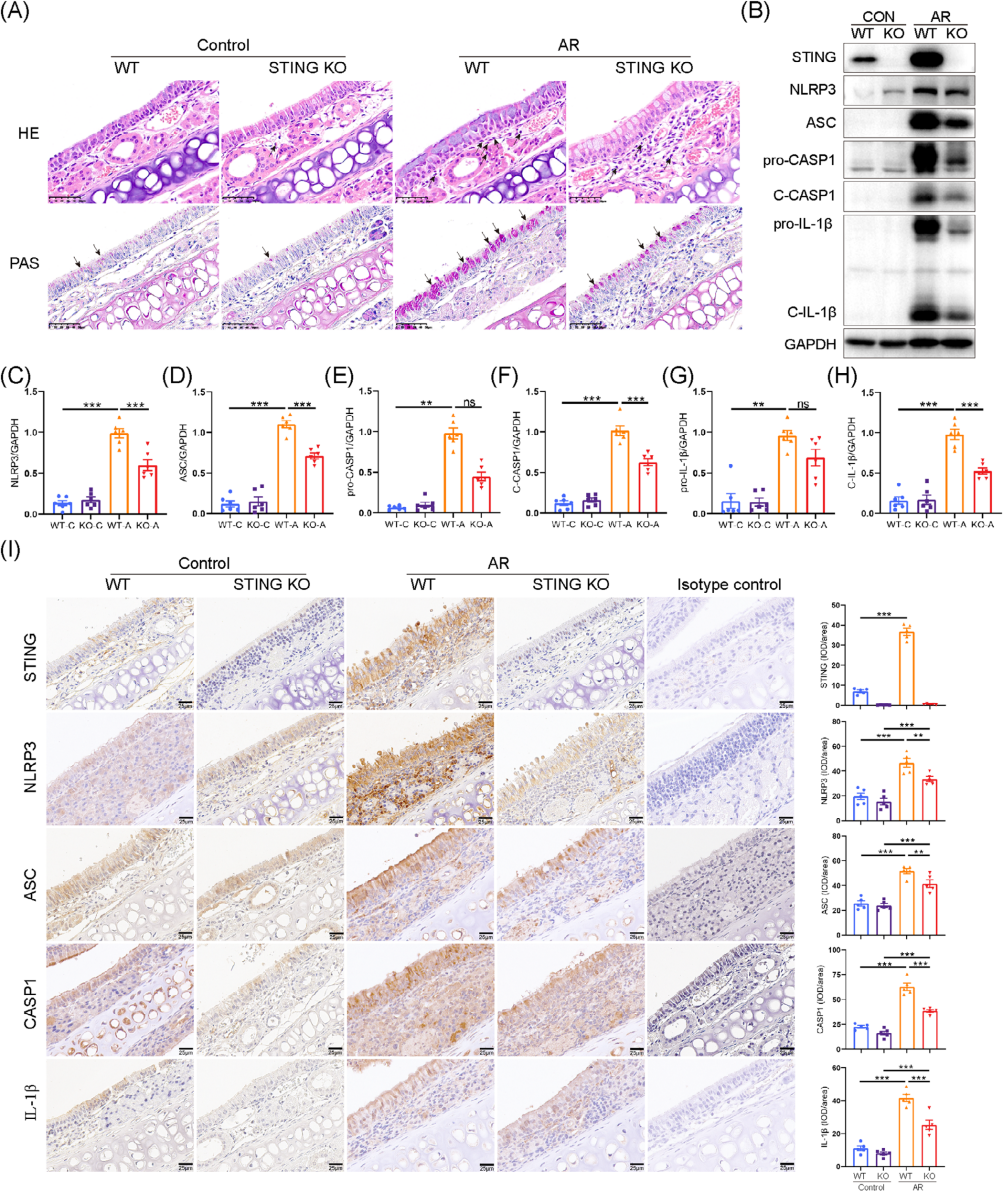
Activation of NLRP3 inflammasome in wild‐type (WT) and STING^–/–^ mice with and without allergic rhinitis (AR). (A) Eosinophil infiltration and goblet cell hyperplasia were evaluated in haematoxylin and eosin (H&E)‐ or PAS‐stained sections of WT and STING^–/–^ mice with and without AR, respectively (*n* = 5; ×400 magnification). (B–H) Expression of protein levels of STING and NLRP3 inflammasome in WT and STING^–/–^ mice with and without AR (*n* = 6). (I) Immunohistochemical staining for STING and NLRP3 inflammasome in nasal tissue of WT and STING^–/–^ mice with and without AR, respectively (*n* = 5; ×400 magnification). *p < .05, **p < .01 and ***p < .001. ns = not significant.

Next, STING knockout mice were used to determine the role of STING signalling in the activation of NLRP3 inflammasome in murine AR model. The knockout efficiency of STING was assessed by Western blotting (Figure S7D). STING deficiency reduced eosinophilic infiltration and goblet cell hyperplasia in the nasal mucosa of AR mice compared to the WT group (Figure 8A). Not surprisingly, STING deficiency significantly inhibited the activation of NLRP3 inflammasome in the nasal mucosa of HDM‐challenged AR mice (Figure 8B–H). The IHC staining analysis consistently yielded results analogous to those obtained through Western blot analysis (Figure 8I). Collectively, these findings indicated that inhibition of STING could alleviate NLRP3 inflammasome activation and disease progression in HDM‐induced AR.

**FIGURE 9:**
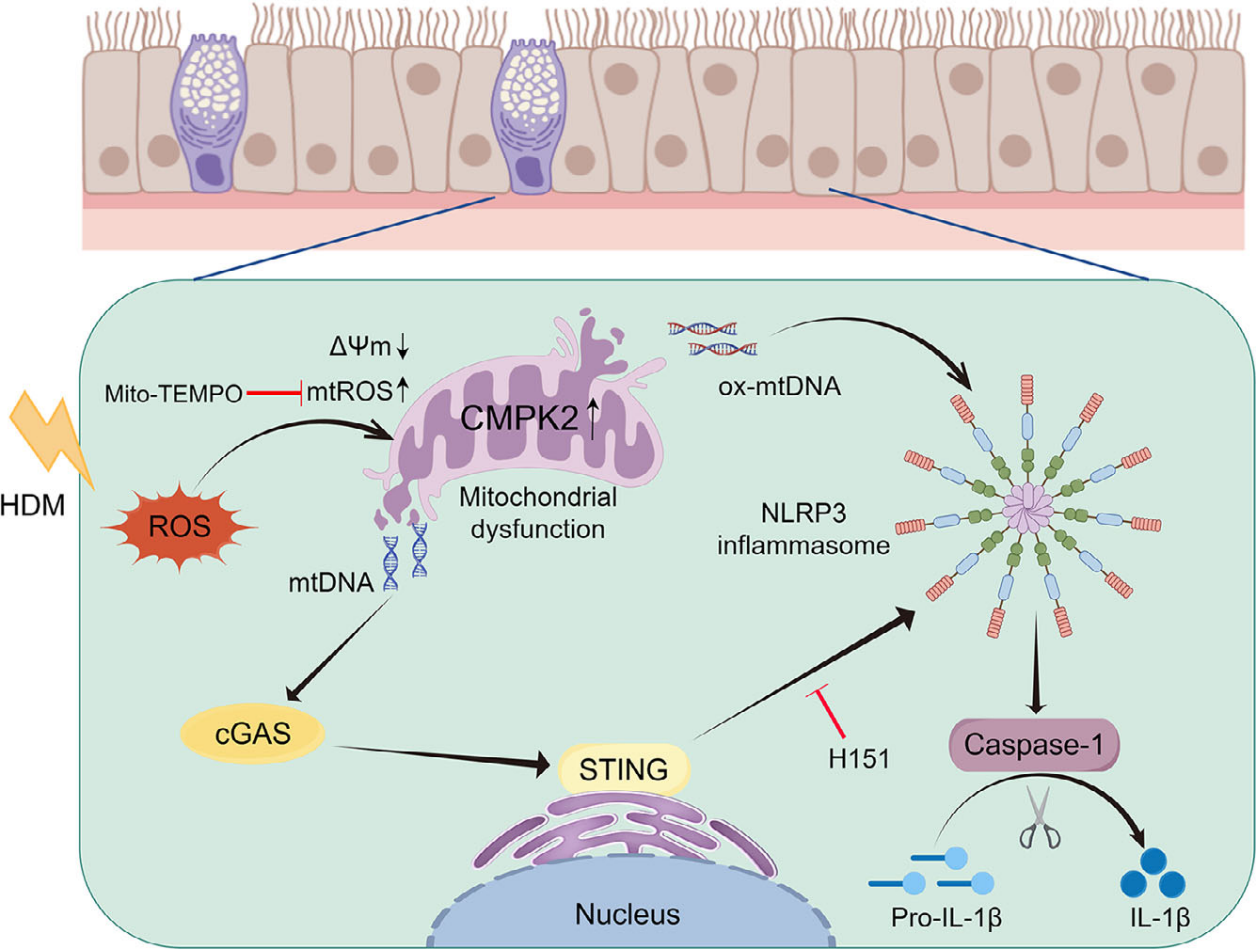
The diagram encapsulates our hypothesis that cytidine/uridine monophosphate kinase 2 (CMPK2) promotes the progression of allergic rhinitis by activating the NLRP3 inflammasome via the mitochondrial DNA (mtDNA)‐STING signalling pathway. Stimulation by house dust mite (HDM) results in the up‐regulation of CMPK2 expression and induces mitochondrial dysfunction, consequently causing the release of mtDNA into the cytoplasm. This release activates the STING signalling pathway, which subsequently leads to the activation of the NLRP3 inflammasome. Inhibition of CMPK2 and the STING signalling pathway can significantly mitigate the activation of the NLRP3 inflammasome, thereby alleviating allergic rhinitis. cGAS, cyclic GMP‐AMP synthase; HDM, house dust mite; ROS, reactive oxygen species.

## 4 DISCUSSION

In this study, we identified the essential role of CMPK2 in regulating NLRP3 inflammasome activation in AR. HDM‐induced mitochondrial dysfunction led to the accumulation of mtROS, cytoplasmic release of mtDNA and oxidation of mtDNA, processes that were primarily dependent on CMPK2 expression. This cascade may trigger NLRP3 inflammasome activation via the mtDNA‐cGAS‐STING axis in AR. Our findings highlight the significant contribution of CMPK2 to the pathogenesis of AR.

Previous studies have shown that HDM‐induced innate immune responses differ between allergic and non‐allergic individuals, but their role in the pathogenesis of AR has remained unclear.^54^, ^55^ Our analysis of GSE9150 in GEO DataSets revealed that expression of haematologic/immune system‐specific hub genes CMPK2 is significantly elevated in allergic samples compared with healthy control samples exposed to HDM. It has been reported that CMPK2 can activate the NLRP3 inflammasome, thereby promoting inflammatory responses in a variety of diseases.^47^, ^48^ In our research, we have identified for the first time that CMPK2 expression is markedly up‐regulated in AR and is correlated with the activation of the NLRP3 inflammasome. Moreover, CMPK2 deficiency significantly inhibited NLRP3 inflammasome activation in HNEPCs. Notably, while the overexpression of CMPK2 markedly enhanced the expression of C‐IL‐1β in HNEPCs stimulated by HDM, its impact on the activation of NLRP3 was comparatively minor. This observation implies the potential involvement of alternative pathways in the regulation of C‐IL‐1B by CMPK2, warranting further investigation. Having identified the crucial roles of CMPK2 in NLRP3 activation, we proceeded to validate our findings through in vivo experiments. The results demonstrated that the elimination of CMPK2 significantly inhibited the expression and activation of NLRP3 inflammasome, thereby alleviating the progression of AR. Overall, these results suggest that NLRP3 activation in AR is CMPK2‐dependent. Nonetheless, the potential existence of additional mechanisms governing CMPK2 expression in airway epithelial cells necessitates further investigation. For instance, exploring the correlation between genetic variants and CMPK2 expression in both allergic and non‐allergic populations represents a promising avenue for future research.

Mitochondria play crucial roles in diverse cellular processes including energy production, intracellular signalling, cell death, ROS generation and immune response.^56^ Several studies have indicated that mtDNA was involved in the activation of NLRP3 inflammasome.^57^ Wu et al. found that Gata6 could regulate the NLRP3 pathway through CMPK2, leading to monocyte recruitment and pro‐inflammatory macrophage formation.^47^ Zhong et al. have shown that CMPK2 deficiency leads to reduced mtDNA synthesis, resulting in inhibition of NLRP3 activation.^26^ In our study, we found that HDM stimulation increases the release of mtDNA to the cytoplasm of HNEPCs, resulting in activation of the NLRP3 inflammasome, which is CMPK2‐dependent. Moreover, we also found that mtDNA depletion reversed HDM‐induced NLRP3 inflammasome activation. These experiments suggest that CMPK2 is involved in the pathogenesis of AR by regulating the activation of NLRP3 inflammasome through mtDNA. However, further investigation is required to determine whether EtBr, in addition to inhibiting mtDNA replication through binding to mtDNA, also contributes to mtDNA depletion by adversely affecting mitochondrial health. Moreover, we also observed that Mito‐TEMPO, a mitochondrial targeting antioxidant, attenuated HDM‐induced activation of the NLRP3 inflammasome, possibly due to the inhibition of CMPK2 expression and STING phosphorylation. Mito‐TEMPO is expected to be a potential therapeutic agent for the treatment of AR.

The cGAS‐STING signalling axis was first discovered to detect pathogenic DNA, eliciting an innate immune response to microbial infections. Notably, recent studies revealed that cGAS‐STING could also be activated by endogenous DNA, which is involved in a number of inflammation‐related diseases, such as, acute respiratory distress syndrome (ARDS),^58^ and chronic endometritis.^59^ Ouyang et al. found that the cGAS‐STING pathway triggered ocular surface inflammation, and suppression of STING ameliorated inflammatory responses and ocular surface diseases progression.^60^ Cao et al. showed that STING knockout attenuated lipopolysaccharide (LPS)‐induced acute kidney injury through STING/ER stress/mtROS/NLRP3 inflammasome axis.^61^ However, the role of the cGAS‐STING pathway in HDM‐induced AR has not been well investigated. In our study, we found that the cGAS‐STING pathway was enhanced in an in vivo mice model, AR patients and an in vitro cell culture model. Moreover, our findings demonstrated that the activation of NLRP3 could be inhibited by either elimination or pharmacological suppression of STING, suggesting that the STING pathway may be involved in the synthesis of NLRP3 inflammasome‐related components, thereby enhancing and maintaining NLRP3 inflammasome activation. Notably, STING inhibition failed to fully block NLRP3 inflammasome activation, suggesting that other unidentified stimulator(s) may be involved in HDM‐NLRP3 inflammasome activation in AR in addition to the mtDNA/STING/NLRP3 pathway.

Cumulatively, our study demonstrates that CMPK2 expression triggers NLRP3 inflammasome activation through the mtDNA‐STING pathway, which plays a crucial role in the pathogenesis of HDM‐induced AR. Furthermore, we show that NLRP3 inflammasome activation is driven by mitochondrial dysfunction and mtROS generation, a process that can be prevented by Mito‐TEMPO (Figure 9). These findings provide new insights into a potential therapeutic strategy for the treatment of AR.

## AUTHOR CONTRIBUTIONS

YaoMing Zheng, YaDong Xie and JiaYing Li are co‐first authors. Material preparation, data collection and analysis were performed by YaoMing Zheng and YaDong Xie. YaoMing Zheng, YuJie Cao, Min Li, JiaYing Li, Qing Cao, MiaoMiao Han, YiLai Shu and HongFei Lou drafted revised the manuscript. HuaBin Li and Hui Xiao were involved in conceptualisation and funding acquisition. All the authors reviewed, revised and approved the final manuscript.

## CONFLICT OF INTEREST STATEMENT

The authors declare no conflicts of interest.

## ETHICS STATEMENT

The ethics committee of Eye and ENT Hospital, Fudan University, approved this study, and all subjects gave their written informed consent.

## Supporting information



Supporting Information

Supporting Information

Supporting Information

Supporting Information

## Data Availability

The data that support the findings of this study are available on request from the corresponding author.
