# Supplementary material for: CMPK2 promotes NLRP3 inflammasome activation via mtDNA‐STING pathway in house dust mite‐induced allergic rhinitis
Source: Clin Transl Med. 2025 Jan 12;15(1):e70180. doi: 10.1002/ctm2.70180 (PMC11726638; doi:10.1002/ctm2.70180)
Supplement: Supplementary file 1 — Supporting Information [file CTM2-15-e70180-s002.docx]

CMPK2 promotes NLRP3 inflammasome activation via mtDNA-STING pathway in house dust mite-induced allergic rhinitis

**Table S1: Demographic characteristics of enrolled subjects**

|  | **Control** | **AR** |
| --- | --- | --- |
| **Total subjects enrolled** | 30 | 25 |
| **Methodology used** |  |  |
| ***Immunohistochemistry*** |  |  |
| Subject number | 15 | 15 |
| Gender, male | 11 (73%) | 10 (67%) |
| Age (years) | 39 (17-60) | 42 (16-68) |
| Asthma | 0 (0) | 2 (13%) |
| ***Western blot*** |  |  |
| Subject number | 15 | 15 |
| Gender, male | 9 (60%) | 10 (67%) |
| Age (years) | 40 (26-60) | 45 (25-59) |
| Asthma | 0 (0) | 1 (7%) |

AR:allergic rhinitis.

**Table S2. Primary antibodies used in westernblotting, immunohistochemistry and immunofluorescence**

| **Antibody** | **Application** | **Species** | **Concentration** | **Clone ID** | **Reference** | **Source** |
| --- | --- | --- | --- | --- | --- | --- |
| CMPK2 | WB | Rabbit | 1:1000 | polyclonal | PA5-34461 | Thermo Fisher Scientific |
| CMPK2 | IHC,IF | Rabbit | 1:300 | polyclonal | PA5-34461 | Thermo Fisher Scientific |
| NLRP3 | WB | Rabbit | 1:1000 | D4D8T | 15101 | Cell Signaling Technology |
| NLRP3 | IHC, IF | Rabbit | 1:200 | polyclonal | 19771-1-AP | Proteintech |
| ASC | WB | Rabbit | 1:500 | polyclonal | 10500-1-AP | Proteintech |
| ASC | IHC, IF | Rabbit | 1:200 | polyclonal | 10500-1-AP | Proteintech |
| ASC | IHC, IF | Mouse | 1:200 | B-3 | sc-514414 | Santa Cruz Biotechnology |
| Caspase-1  (P20/P10) | WB | Rabbit | 1:1000 | polyclonal | 22915-1-AP | Proteintech |
| Caspase-1 | WB | Mouse | 1:500 | 14F468 | sc-56036 | Santa Cruz Biotechnology |
| Caspase-1 | WB | Rabbit | 1:1000 | EPR19672 | ab207802 | Abcam |
| Caspase-1  (P20/P10) | IHC, IF | Rabbit | 1:300 | polyclonal | 22915-1-AP | Proteintech |
| IL-1β | IHC | Rabbit | 1:100 | 3A6 | 12242 | Cell Signaling Technology |
| IL-1β | IHC | Rabbit | 1:200 | polyclonal | P50520-1R1 | Abmart |
| IL-1β | WB | Rabbit | 1:1000 | 3A6 | 12242 | Cell Signaling Technology |
| STING | IHC | Rabbit | 1:300 | D2P2F | 13647 | Cell Signaling Technology |
| STING | IF | Mouse | 1:200 | AG20363 | 66680-1-Ig | Proteintech |
| P-STING | WB | Rabbit | 1:1000 | D8F4W | 72971 | Cell Signaling Technology |
| P-STING | WB | Rabbit | 1:1000 | D7C3S | 19781 | Cell Signaling Technology |
| cGAS | WB | Rabbit | 1:1000 | D3O8O | 31659 | Cell Signaling Technology |
| cGAS | WB | Rabbit | 1:1000 | E5V3W | 79978 | Cell Signaling Technology |
| GAPDH | WB | Rabbit | 1:5000 | AG0766 | 60004-1-Ig | Proteintech |
| 8-OHdG | IF | Mouse | 1:100 | E-8 | sc-393871 | Santa Cruz Biotechnology |
| dsDNA | IF | Mouse | 1:100 | HYB331-01 | sc-58749 | Santa Cruz Biotechnology |
| ACTIN | WB | Rabbit | 1:2000 | polyclonal | [P30002](http://www.ab-mart.com.cn/page.aspx?node= 59 &id= 1193" \t "https://www.ab-mart.com.cn/_blank) | Abmart |
| β-Tubulin | WB | Mouse | 1:2000 | 2H4 | M30109 | Abmart |

CMPK2, Cytidine/uridine monophosphate kinase 2, NLRP3, NOD-like receptor family, pyrin domain containing 3; ASC，apoptosis-associated speck-like protein containing a caspase-recruitment domain; IL-1β, interleukin 1β; STING, stimulator of interferon genes; p-STING, phosphorylated STING; cGAS, Cyclic GMP-AMP synthase; WB, western blot; IHC, immunohistochemistry; IF, immunofluorescence.

**Table S3. Primers used for quantitative RT-PCR analysis.**

| **Primer** | **Sequence** | **Annealing temperature (℃)** | **Expect product**  **size (bp)** |
| --- | --- | --- | --- |
| NLRP3 | (F)5’-GATCTTCGCTGCGATCAACAG-3’ | 60 | 81 |
|  | (R)5’-CGTGCATTATCTGAACCCCAC-3’ |  |  |
| CASP1 | (F)5’-TTTCCGCAAGGTTCGATTTTCA-3’ | 60 | 54 |
|  | (R)5’-GGCATCTGCGCTCTACCATC-3’ |  |  |
| ASC | (F)5’-TGGATGCTCTGTACGGGAAG-3’ | 60 | 110 |
|  | (R)5’-CCAGGCTGGTGTGAAACTGAA-3’ |  |  |
| IL-1β | (F)5’-ATGATGGCTTATTACAGTGGCAA-3’ | 59 | 132 |
|  | (R)5’-GTCGGAGATTCGTAGCTGGA-3’ |  |  |
| GAPDH | (F)5’-GAGTCAACGGATTTGGTCGT-3’ | 58 | 184 |
|  | (R)5’-TTGATTTTGGAGGGATCTCG-3’ |  |  |
| Periostin | (F)5’-GTCTTTGAGACGCTGGAAGG-3’ | 58 | 203 |
|  | (R)5’-AGATCCGTGAAGGTGGTTTG-3’ |  |  |
| CSF2 | (F)5’-TCCTGAACCTGAGTAGAGACAC -3’ | 60 | 187 |
|  | (R)5’-TGCTGCTTGTAGTGGCTGG -3’ |  |  |
| IL-6 | (F)5’-AGTAGTGAGGAAACAAGCCAGAGC-3’ | 62 | 107 |
|  | (R)5’-TTGGGTCAGGGGTGGTTATTG-3’ |  |  |
| IL-8 | (F)5’-ACTGAGAGTGATTGAGAGTGGAC-3’ | 60 | 112 |
|  | (R)5’-AACCCTCTGCACCCAGTTTTC-3’ |  |  |
| CCL5 | (F)5’-TACACCAGTGGCAAGTGCTC -3’ | 60 | 199 |
|  | (R)5’-GAAGCCTCCCAAGCTAGGAC -3’ |  |  |
| CMPK2 | (F)5’-GTACCTCCTTTATTCCTGAAGCC -3’ | 59 | 94 |
|  | (R)5’-ATGGCAACAACCTGGAACTTT -3’ |  |  |
| IFI44 | (F)5’-TTTGCTCTTTCTGACATCTCGGT -3’ | 60 | 164 |
|  | (R)5’-TCCTCCCTTAGATTCCCTATTTGC -3’ |  |  |
| RSAD2 | (F)5’-TTGGACATTCTCGCTATCTCCT-3’ | 59 | 203 |
|  | (R)5’-AGTGCTTTGATCTGTTCCGTC-3’ |  |  |
| RRM1 | (F)5’-CTTGCCCAGACTCAACAT-3’ | 56 | 185 |
|  | (R)5’-CCAGACAGCACTTTCTTCAG-3’ |  |  |

**Table S4. siRNA sequence.**

| **siRNA primer** | **Sense** | **Antisense** |
| --- | --- | --- |
| human-siRNA-STING-1 | 5'-GCCCGGAUUCGAACUUACAAUTT-3' | 5'-AUUGUAAGUUCGAAUCCGGGCTT-3' |
| human-siRNA-STING-2 | 5'-GCUGGCAUGGUCAUAUUACAUTT-3' | 5'-AUGUAAUAUGACCAUGCCAGCTT-3' |
| human-siRNA-STING-3 | 5'-GUUUACAGCAACAGCAUCUAUTT-3' | 5'-AUAGAUGCUGUUGCUGUAAACTT-3' |
| human-siRNA-CMPK2-1 | 5'-CAGUGGCAGAUUCACUUAATT-3' | 5'-UUAAGUGAAUCUGCCACUGTT-3' |
| human-siRNA-CMPK2-2 | 5'-CGAAAUAGCUAAAGAAUCUTT-3' | 5'-AGAUUCUUUAGCUAUUUCGTT-3' |
| human-siRNA-CMPK2-3 | 5'-CCAACAGUGUGUUUCGUCATT-3' | 5'-UGACGAAACACACUGUUGGTT-3' |
| Negative control | 5'-UUCUCCGAACGUGUCACGUTT-3' | 5'-ACGUGACACGUUCGGAGAATT-3' |
| FAM Negative control | 5'-UUCUCCGAACGUGUCACGUTT-3' | 5'-ACGUGACACGUUCGGAGAATT-3' |
| Positive control (GAPDH) | 5'- UGACCUCAACUACAUGGUUTT-3' | 5'-AACCAUGUAGUUGAGGUCATT-3' |

**Table S5. mtDNA Primers used for quantitative RT-PCR analysis.**

| **Primer** | **Sequence** |
| --- | --- |
| ND1 | (F)5’-CACCCAAGAACAGGGTTTGT-3’ |
|  | (R)5’-TGGCCATGGGTATGTTGTTAA-3’ |
| D-LOOP | (F)5’-CTATCACCCTATTAACCACTCA-3’ |
|  | (R)5’-TTCGCCTGTAATATTGAACGTA-3’ |
| ATP6 | (F)5’-CCCTGGCCGTACGCCTAACC-3’ |
|  | (R)5’-GCTAGGGTGGCGCTTCCAAT-3’ |
| 18S | (F)5’-TAGAGGGACAAGTGGCGTTC-3’ |
|  | (R)5’-CGCTGAGCCAGTCAGTGT-3’ |

**Table S6. Distribution of tissue/organ-specific expressed genes identified by BioGPS.**

| System/Organ | Genes | Counts |
| --- | --- | --- |
| Hematologic/immune | MRPL16, POP5, ANP32E, B3GNT2, DCPS, NTPCR, HDDC3, EAF2, DUSP5, THEMIS2, SP140L, EBAG9, RGS2, HACD4, TRIB1, IFI44, TNFAIP2, IER2, MRPL1, TMEM218, PAG1, RESF1, ADAM8, SPATA13, CCL5, PPP1R18, METRNL, CHST2, RRM1, IL32, ID2, HSP70B, RSAD2, CMPK2, TRIM6-TRIM34, TLR1, ZNF267, BACH2 | 38 |
| Nervous | ENC1, GNAL, FGF13, MLLT11, MN1, TPPP, QDPR, MPI, NUAK1, RGS4, PDP1, CAMK2N1, CDK5 | 13 |
| Digestive | SLC26A2, ST6GALNAC1, MSX1, GC, CYP4F11, SLC5A1, MAF, AKR1B10, TMEM45B | 9 |
| Skeletal/Smooth Muscle | GPX8, TGFB2, RRAD, MMP1, PXDN | 5 |
| Placenta | PSG4, HSD17B2, IL1RL1, KISS1, TIMP2 | 5 |
| Genital | IL13RA2, SPA17, HSPA4L, MMP10, PRPS1L1 | 5 |
| Respiratory | LAMB3, LAMC2, PNPLA4, CYP1A1 | 4 |
| Cardiac Myocytes | STC2, F2R | 2 |
| Tongue | MAL, KRT13 | 2 |

**Table S7. Top 20 hub genes based on MCC score.**

| **Gene Symbol** | **Full Name** | **MCC Score** | **logFC** | **adj.P-value** |
| --- | --- | --- | --- | --- |
| RFC4 | replication factor C subunit 4 | 123315 | -2.70749 | 1.19E-05 |
| CCNB1 | cyclin B1 | 123292 | -2.38174 | 0.00044 |
| RFC3 | replication factor C subunit 3 | 122606 | -2.18184 | 5.91E-06 |
| MCM6 | minichromosome maintenance complex component 6 | 122568 | -2.54401 | 3.17E-06 |
| RRM2 | ribonucleotide reductase regulatory subunit M2 | 122504 | -2.73326 | 0.00802 |
| GMNN | geminin DNA replication inhibitor | 122449 | -2.40689 | 3.68E-05 |
| PBK | PDZ binding kinase | 122400 | -2.05065 | 0.00985 |
| ZWINT | ZW10 interacting kinetochore protein | 81363 | -2.66402 | 3.84E-06 |
| RRM1 | ribonucleotide reductase catalytic subunit M1 | 80732 | -2.12324 | 9.26E-05 |
| ISG15 | ISG15 ubiquitin like modifier | 41294 | 2.06478 | 0.02111 |
| OASL | 2'-5'-oligoadenylate synthetase like | 41286 | 2.41042 | 0.00157 |
| RSAD2 | radical S-adenosyl methionine domain containing 2 | 41280 | 3.70809 | 0.00036 |
| DDX60 | DExD/H-box helicase 60 | 41160 | 4.41188 | 2.47E-05 |
| TIPIN | TIMELESS interacting protein | 41056 | -3.08092 | 1.41E-05 |
| IFI44 | interferon induced protein 44 | 41040 | 2.30993 | 0.00365 |
| RTP4 | receptor transporter protein 4 | 41040 | 3.21793 | 4.30E-05 |
| DDX58 | DExD/H-box helicase 58 | 40600 | 2.12632 | 0.00032 |
| UBE2T | ubiquitin conjugating enzyme E2 T | 40445 | -2.37440 | 0.00014 |
| CMPK2 | cytidine/uridine monophosphate kinase 2 | 40329 | 3.55757 | 0.00014 |
| HERC5 | HECT and RLD domain containing E3 ubiquitin protein ligase 5 | 40320 | 2.95022 | 0.00015 |
