# Supplementary material for: CMPK2 promotes NLRP3 inflammasome activation via mtDNA‐STING pathway in house dust mite‐induced allergic rhinitis
Source: Clin Transl Med. 2025 Jan 12;15(1):e70180. doi: 10.1002/ctm2.70180 (PMC11726638; doi:10.1002/ctm2.70180)
Supplement: Supplementary file 2 — Supporting Information [file CTM2-15-e70180-s004.docx]

CMPK2 promotes NLRP3 inflammasome activation via mtDNA-STING pathway in house dust mite-induced allergic rhinitis


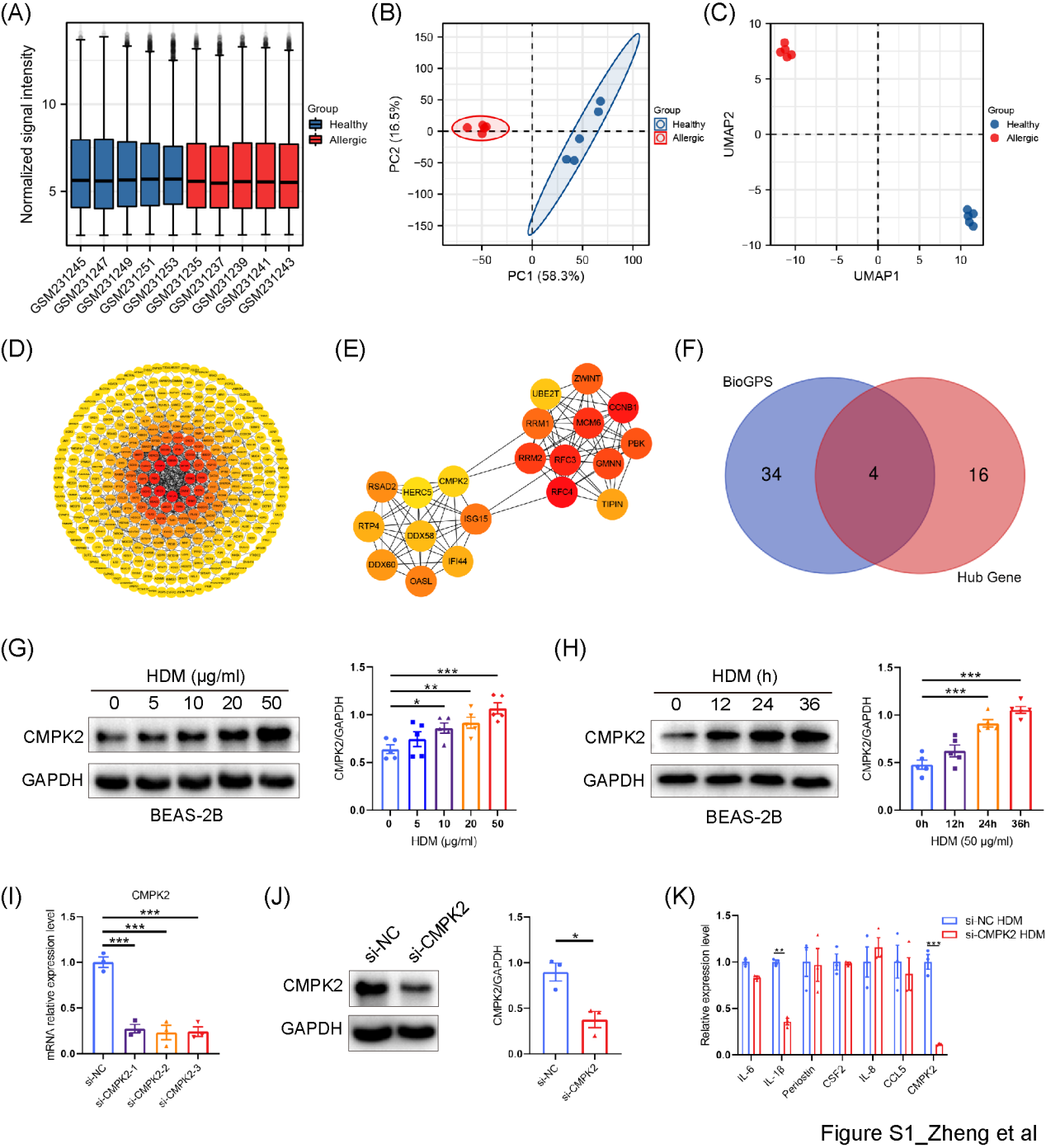


**Figure S1: Identification of DEGs.**

1. Box plot of the normalized gene expression profiles. (B) PCA between allergic and healthy groups on gene expression profiles. (C) UMAP shows significant segregation of the two groups. (D) PPI network of DEGs**.** (E) Identification of the hub genes through maximal clique centrality (MCC) algorithm. The red nodes represent genes with a high MCC sore, and the yellow ones represent genes with a low MCC sore. (F) Venn diagram of system-specific expressed hub genes between the top 20 hub genes and tissue/organ-specific expressed genes. (G) BEAS-2B cells were stimulated with HDM at the indicated concentration for 24 hours, CMPK2 protein levels were measured by Western blotting (n = 5). (H) BEAS-2B cells were stimulated with HDM (50μg/ml) at the indicated hours. CMPK2 protein levels were measured by Western blotting (n = 5). (I) Expression of mRNA levels of CMPK2 was assessed by quantitative PCR in HNEPC cells after si-CMPK2 transfection. (J) Western blotting analysis of CMPK2 in HNEPC cells transfected with si-CMPK2. (K) mRNA levels of IL-6, IL-1β, periostin, CSF2, IL-8, CCL5 and CMPK2 in HNEPC cells. *P < .05, **P < .01 and ***P < .001. PPI, protein–protein interaction; DEG, differentially expressed gene. PCA, Principal Component Analysis. UMAP, uniform manifold approximation and projection.


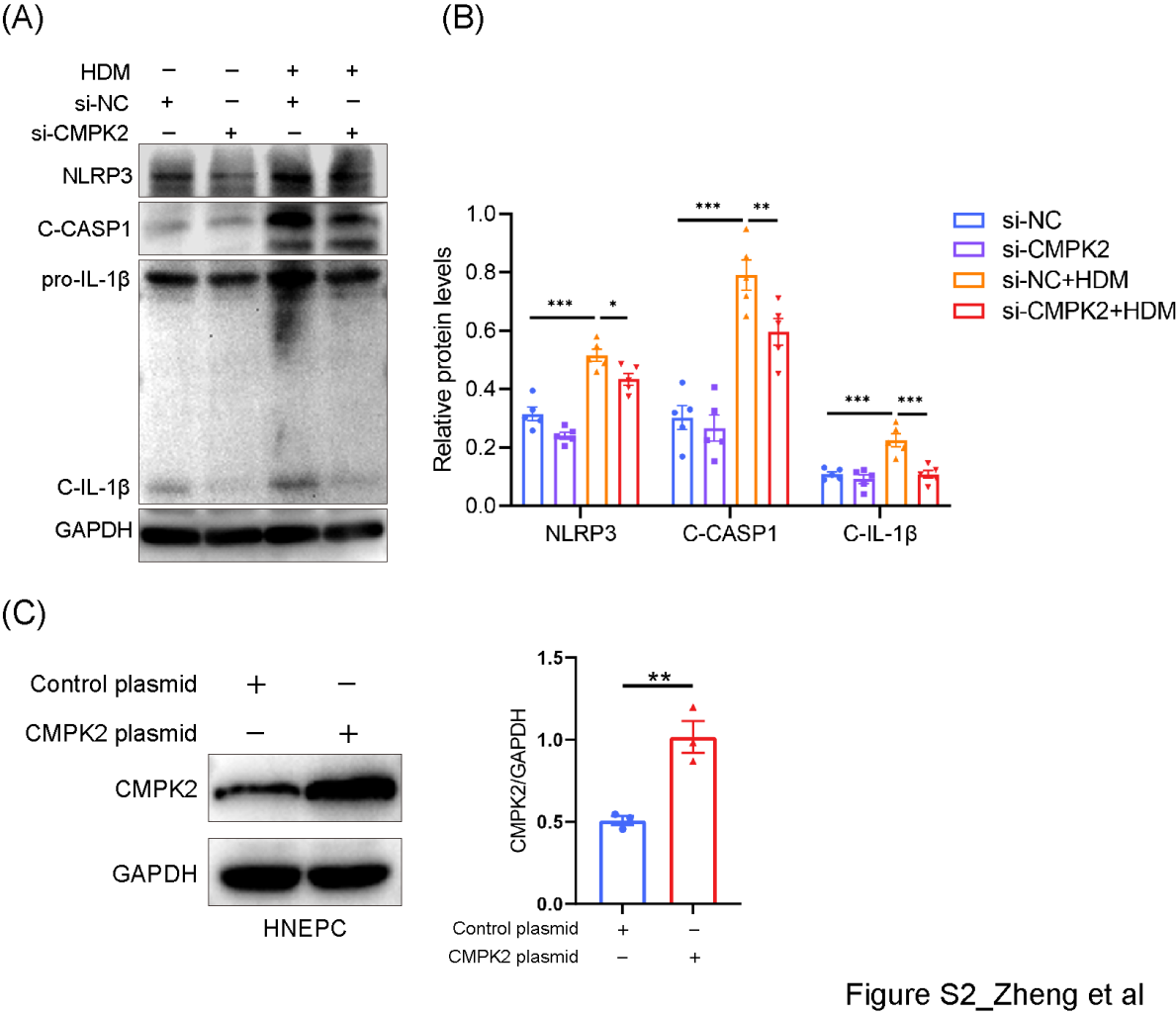


**Figure S2: Downregulated CMPK2 expression inhibits NLRP3 inflammasome activation in HNEPC cells.**

(A-B) HNEPC cells were transfected with si-CMPK2 or control siRNA for 24 hours and further stimulated with HDM (50 μg/mL) for another 24 hours. Cells were collected for Western blotting (n = 5). Representative blots are shown. (C) HNEPC cells were transfected with CMPK2 plasmid for 48 hours. Cell lysates were subjected to Western blotting (n = 3). Representative blots are shown, and densitometric analysis of blots was performed. *P < .05, **P < .01 and ***P < .001.


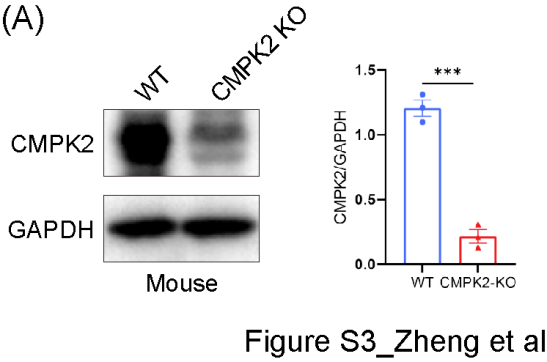


**Figure S3: Validation of CMPK2 knockout mice.**

(A) The CMPK2 protein levels in the nasal tissues of WT and CMPK2^–/–^ mice (n = 3).


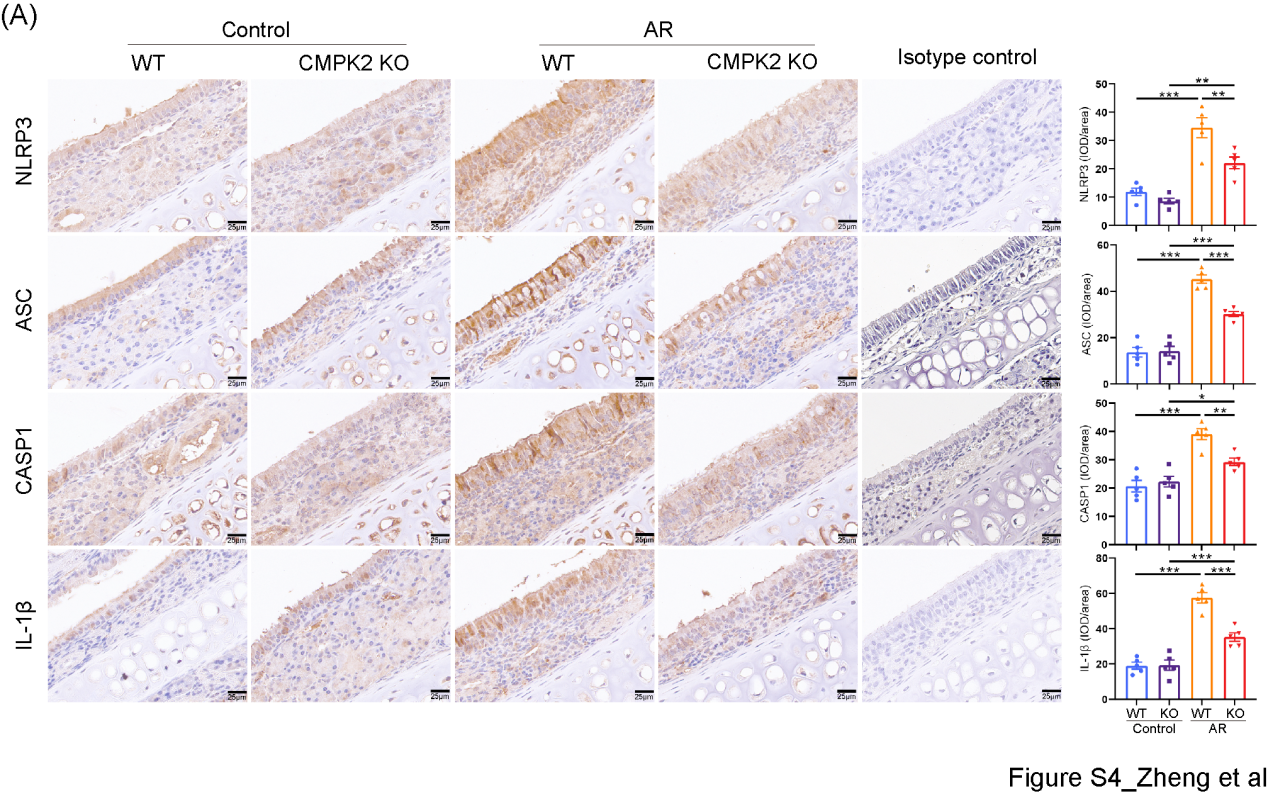


**Figure S4: IHC staining expression of NLRP3 inflammasome in WT and CMPK2^–/–^ mice with and without AR.**

1. Immunohistochemical staining for NLRP3 inflammasome in nasal tissue of WT and CMPK2^–/–^ mice with and without AR, respectively (n = 5) (×400 magnification). *P < .05, **P < .01 and ***P < .001.


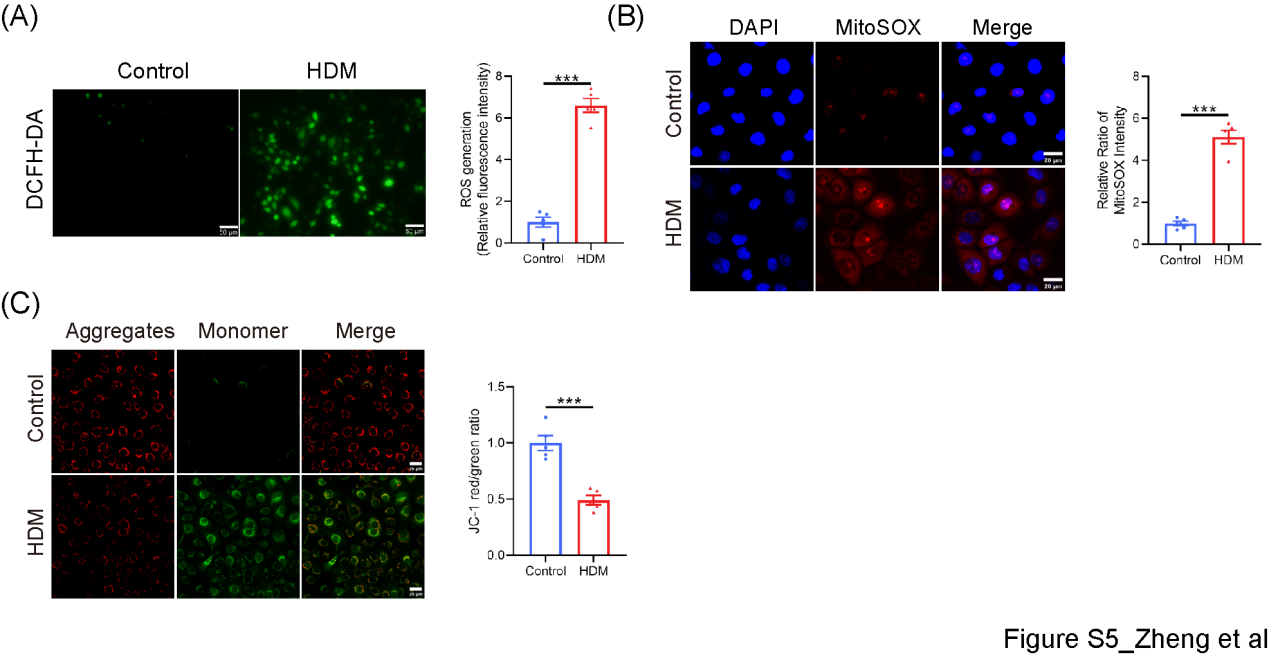


**Figure S5: HDM activates the NLRP3 inflammasome by inducing mitochondrial dysfunction. HNEPC cells stimulated with HDM (50 μg/mL) for 24 hours.**

1. ROS generation after labeling with DCFH-DA (n = 5). Scale bar = 50 μm. (B) Mitochondrial superoxide was evaluated by MitoSOX Red staining in HNEPC cells after stimulation with HDM (50 μg/ml, 24 h) (n = 5). Scale bar = 20 μm. (C) Representative fluorescence images of JC-1 staining in HNEPC cells (n = 5). Scale bar = 25 μm; red, aggregates; green, monomers.


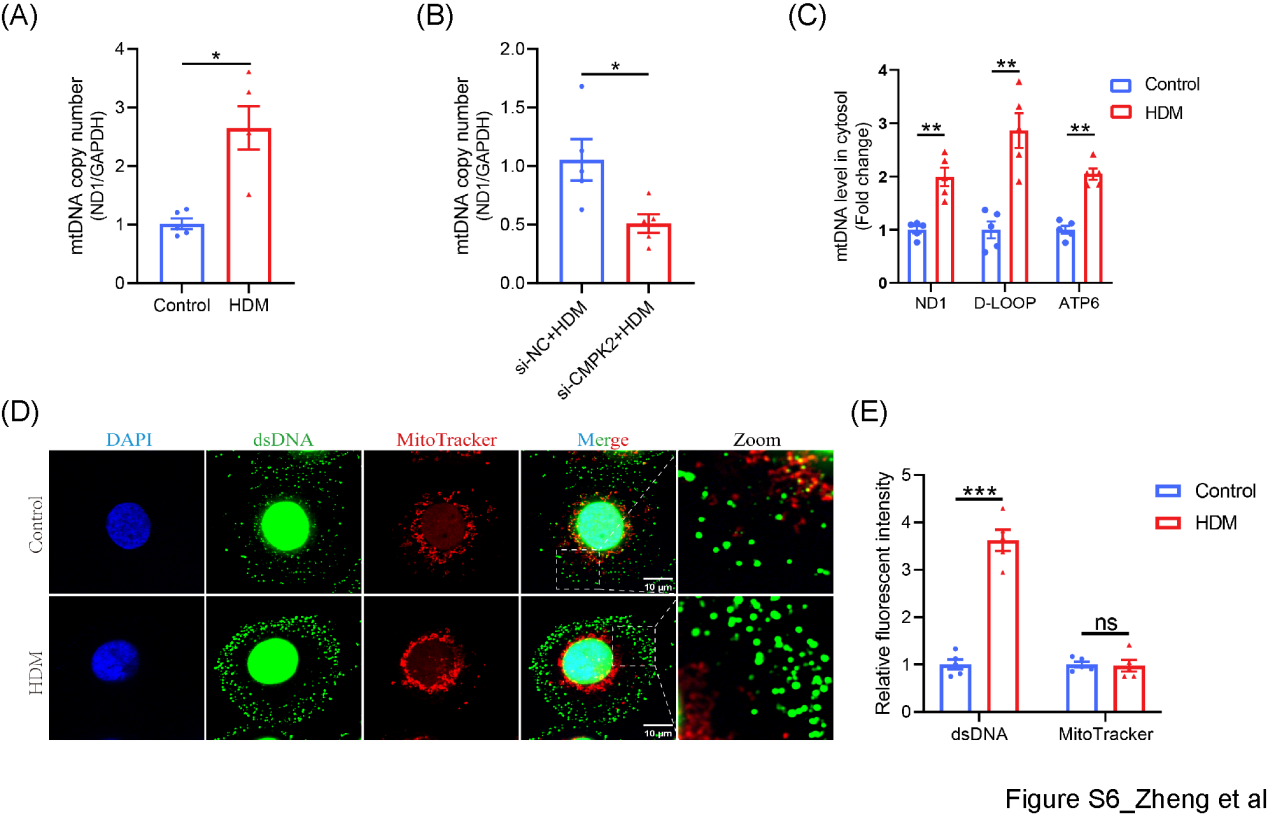


**Figure S6: HDM induced mtDNA leakage into the cytosol in HNEPC cells.**

1. mtDNA copy number was assessed by quantitative PCR in HNEPC cells after treatment with the HDM (50 μg/ml) for 24 h (n = 5). (B) After si-CMPK2 transfection, HNEPC cells were treated with HDM (50 μg/mL) for 24 hours. The mtDNA copy number was assessed by quantitative PCR in HNEPC cells (n = 5). (C) mtDNA levels in the cytoplasm was assessed by quantitative PCR in HNEPC cells after HDM stimulation (50 μg/ml) for 24 h (n = 5). (D-E) Immunofluorescent double-labeling of DNA and mitochondria in HNEPC cells after stimulation with HDM (50 μg/ml) for 24 h was observed by confocal microscopy (n = 5). Double-stranded DNA was stained with anti-dsDNA antibodies (green). Mitochondria was stained with MitoTracker (red). Scale bar = 10 μm. *P < .05, **P < .01 and ***P < .001.


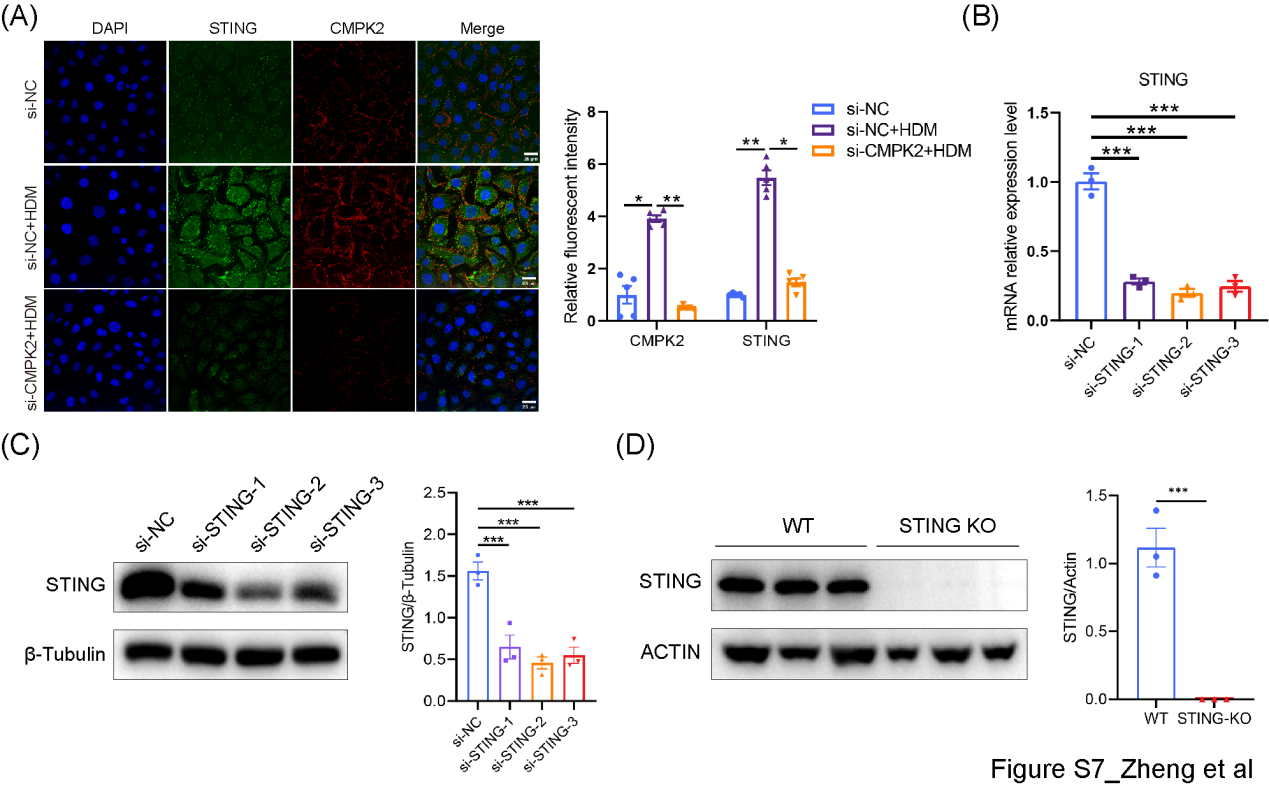


**Figure S7: CMPK2 regulates the activation of STING pathway.**

(A) Representative IF staining of CMPK2 and STING (n = 5). Scale bar = 25 μm. (B) After si-STING transfection for 24 hours, HNEPC cells were collected for RT-PCR (n = 3). (C) HNEPC cells were transfected with si-STING for 48 hours. Cell lysates were subjected to Western blotting (n = 3). (D) Representative blots of STING in the nasal tissues of WT and STING^–/–^ mice (n = 3). *P < .05, **P < .01 and ***P < .001.


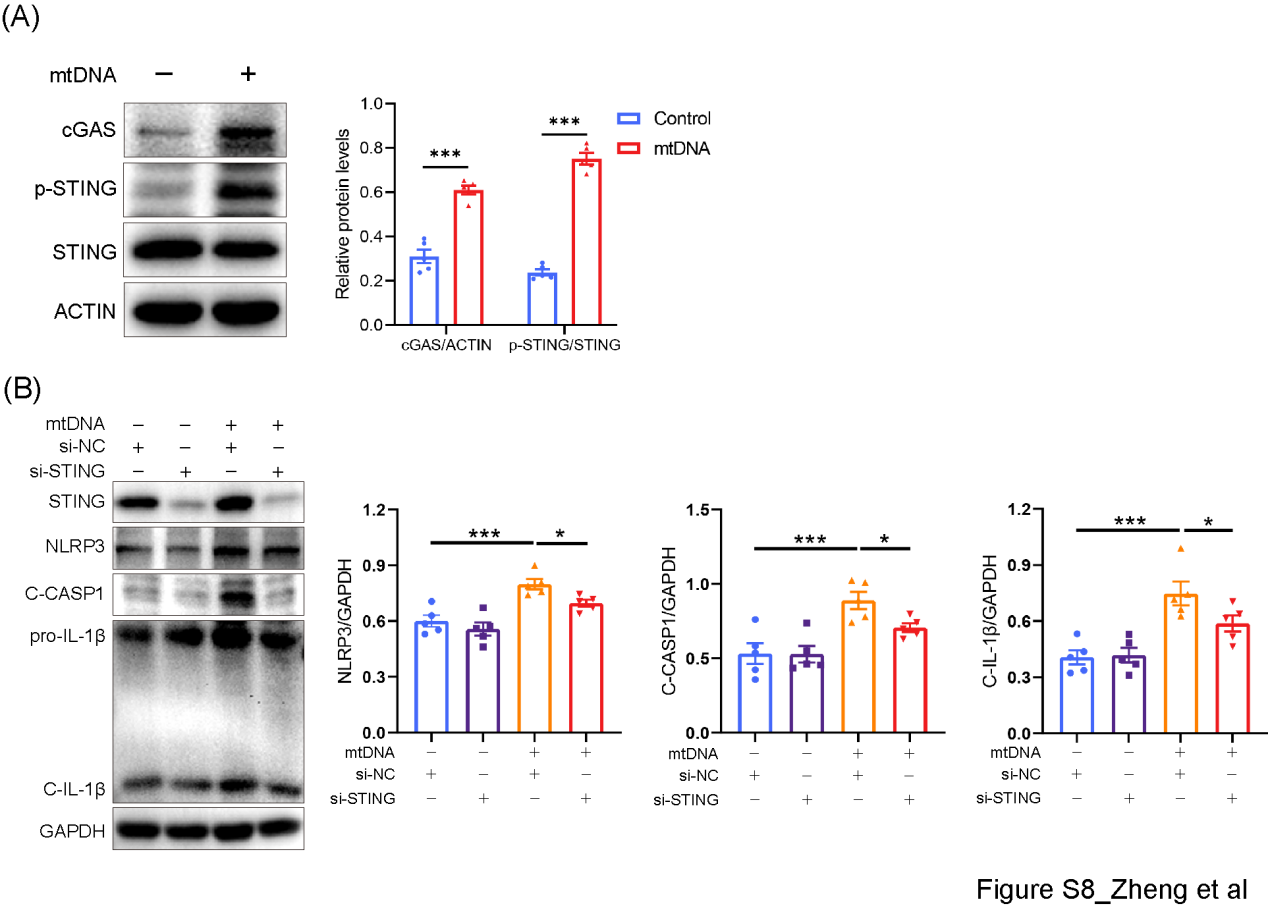


**Figure S8: mtDNA mediates NLRP3 inflammasome activation via the cGAS-STING pathway.**

1. HNEPC cells were transfected with si-STING for 24 hours and further transfected with mtDNA (1 μg/mL) for another 24 hours. The expression of STING and NLRP3 inflammasome in HNEPC cells was determined by Western blotting (n = 5). *P < .05, **P < .01 and ***P < .001.


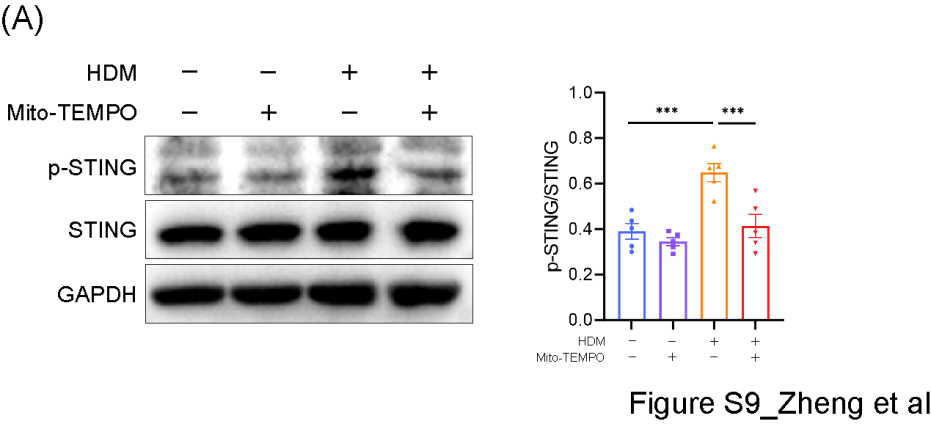


**Figure S9: Mito-TEMPO can inhibit STING phosphorylation induced by HDM.**

1. Representative blots of P-STING and STING expression in HNEPC cells treated with HDM and Mito-TEMPO (n = 5).
