# Supplementary material for: CMPK2 promotes NLRP3 inflammasome activation via mtDNA‐STING pathway in house dust mite‐induced allergic rhinitis
Source: Clin Transl Med. 2025 Jan 12;15(1):e70180. doi: 10.1002/ctm2.70180 (PMC11726638; doi:10.1002/ctm2.70180)
Supplement: Supplementary file 3 — Supporting Information [file CTM2-15-e70180-s001.docx]

CMPK2 promotes NLRP3 inflammasome activation via mtDNA-STING pathway in house dust mite-induced allergic rhinitis

**MATERIALS AND METHODS**

**Subjects**

This study was approved by the ethics committee of Eye and ENT Hospital, Fudan University, and written informed consent was obtained from all the subjects. A total of 30 control subjects, 25 patients with allergic rhinitis (AR) were included in this study. The diagnosis of AR was made according to the 2018 International Consensus Statement on Allergy and Rhinology: Allergic Rhinitis (ICAR-Allergic Rhinitis 2018)^1^. All AR patients are allergic to house dust mites. Inferior turbinates were obtained from AR patients who underwent surgical intervention for septoplasty or nasal obstruction. As normal controls, 30 non-atopic patients undergoing non-sinonasal-related surgeries because of nasal septum deviation, cerebrospinal fluid rhinorrhea, and anterior skull base tumors but did not have any other nasal and sinus disease were enrolled. The patients who received [allergen immunotherapy](https://www.sciencedirect.com/topics/immunology-and-microbiology/allergen-immunotherapy), diagnosed with infectious diseases, pregnant, or treated with concomitant medications (such as [antihistamines](https://www.sciencedirect.com/topics/pharmacology-toxicology-and-pharmaceutical-science/antihistaminic-agent), steroids, and immune drugs) were excluded. Not all samples were included in every study protocol because of the limit of tissues. More information is provided in [Table S1](https://www.sciencedirect.com/science/article/pii/S009167492032409X?via=ihub#appsec1).

**Microarray data acquisition**

GSE9150 was downloaded from the GEO (http://www.ncbi.nlm.nih.gov/geo) database through the GEOquery package (version 2.54.1) in R software (version 3.6.3)^2^. GSE9150 included primary nasal epithelial cells exposed to house dust mite from 5 monotypic HDM-allergic patients and 5 healthy controls and was studied with the GPL570 [HG-U133_Plus_2] Affymetrix Human Genome U133 Plus 2.0 Array.

**Data normalization and identification of DEGs**

Repeated probes for the same gene were eliminated by retaining the probe with the maximum signal value, and the gene matrix had been normalized (supplementary Figure S1). Principal component analysis (PCA) and Uniform Manifold Approximation and Projection (UMAP) was performed to assess the similarity of gene expression patterns between the two groups, and then differentially expressed genes (DEGs) in the GSE9150 microarray were screened using the limma package (version 3.42.2) in R software (adj. p< 0.05, |logFC|>2)^3^. To better visualize these DEGs, volcano plot and heatmap were made by ggplot2 (version 3.3.3) and ComplexHeatmap (version 2.2.0) package, respectively^4^.

**PPI Network Construction and Hub Gene Exploration**

The protein-protein interaction (PPI) network was constructed based on the DEGs by the online tool STRING (version 11.5) (https://string-db.org/) with a filter condition (combined score > 0.4)^5^. Then, we visualized the PPI network by the Cytoscape software (version 3.9.1)^6^. In addition, we used the maximal clique centrality (MCC) algorithm with the Cytohubba plugin to identify hub genes in the PPI network^7^.

**Identification of tissue/organ-specific expressed genes**

To identify the tissue/organ-specific expression of the DEGs, the online resource BioGPS (http://biogps.org/) was used to analyze the tissue distribution^8^. The screening criteria were as follows: (1) the tissue-specific expression level was >10 times the median, and (2) the second highest expression level was less than one-third as high as the highest level^9^. Genes that meet these criteria were deemed to be tissue-specific genes.

**Cell culture**

The BEAS-2B cell was cultured in DMEM media supplemented with 10% FBS, 1% penicillin (100 IU/mL)/streptomycin (100 μg/mL). Human nasal epithelial cells (HNEpCs) were purchased from PromoCell and grown in minimal essential medium supplemented with 10% fetal bovine serum and Antibiotic–Antimycotic (1:100, Gibco, NY, USA)^10^. When a confluence of 80–90% was reached, the cells were treated with house dust mite (HDM, Greer Laboratories, Lenoir, NC). In some experiments, cells were pre-incubated with Mito-TEMPO (Selleck, China) for 3 h. The culture medium was replaced every 48 hours.

**HE staining**

Samples were stained with hematoxylin and eosin (HE) routinely. The mice were euthanized through cervical dislocation. Fresh murine head specimens were fixed overnight with 4% paraformaldehyde, decalcified in EDTA, embedded in paraffin, dewaxed, rehydrated, and used for hematoxylin and eosin (H&E) staining analyses.

**Immunohistochemical (IHC) staining**

IHC was conducted as previously described^11^. The presence of CMPK2, STING, and NLRP3 inflammasome-related proteins was assessed in freshly obtained sinonasal mucosa tissues from murine and patients by immunohistochemistry (IHC). Briefly, all samples were fixed with 4% paraformaldehyde (pH 7.4), embedded in paraffin, and cut into 3-μm-thick sections with a Leica microtome (Leica, Wetzlar, Germany). Heat-induced antigen retrieval was performed in a 0.01 M citrate buffer (pH 6.0) or EDTA buffer (pH 9.0) by microwave for 15 min. The 3% hydrogen peroxide (Sangon Biotechnology, Shanghai, China) was used to quench endogenous peroxidase activity and 5% normal serum of secondary antibody was used to block nonspeciﬁc binding. Sections were then incubated with primary antibodies shown in Table S2 overnight at 4°C, and negative controls were prepared using PBS in place of the primary antibodies. Next, sections were incubated with sufficient peroxidase labeled polymersecondary antibody for 30min at room temperature. Staining was visualized with DAB substrate (Vector Laboratories) and counterstained with hematoxylin. The average optical density (AOD) of per stained area (IOD/area) was calculated by using Image J analysis software.

**Western Blotting**

Tissues and airway epithelial cell lines (HNEPC and BEAS-2B) were lysed in RIPA lysis buffer supplemented with protease inhibitor cocktail (Roche) and phosphatase inhibitors (Beyotime Biotechnology, Beijing, China). Proteins were then separated by 10–12% SDS-PAGE and transferred to a PVDF membrane (Merck Millipore, Darmstadt, Germany). After blocked with 5% non-fat milk at room temperature for 1 hour, the membranes were incubated with primary antibodies described in Table S2 overnight at 4°C. Subsequently, the membrane was incubated with a secondary antibody for another 1 hour. Finally, membranes were detected by using the chemiluminescence reagent. Images were visualized by Tanon image system (Tanon Technology, Shanghai, China).

**Immunofluorescence staining (IF)**

Immunofluorescence staining for cultured HNEPC cells was performed as previously described^12^. Briefly, after cultured, HNEPC cells were fixed with fresh 4% paraformaldehyde for 20 minutes at room temperature. After washing with PBS, cells were permeabilized with 0.2% Triton X-100 in 1X PBS for 5 minutes. Next, fixed and permeabilized cells were blocked with 5% normal serum of secondary antibody in PBS for 1 hour at room temperature and incubated with primary antibodies described in Table S2 at 4℃ overnight. Negative controls were incubated with PBS instead of the primary antibodies. Alexa Fluor 488-conjugated donkey anti-mouse IgG and Alexa Fluor 555-conjugated donkey anti-rabbit IgG (Invitrogen) were subsequently used at recommended concentrations for the secondary antibodies, and cell nuclei were stained with 4ʹ-6-diamidino-2-phenylindole, dihydrochloride (DAPI; Invitrogen). Images were taken under immunofluorescence microscope.

**Quantitative PCR**

Quantitative RT-PCR was performed as stated elsewhere^11,12^. Total RNA was isolated from HNEPC by using a Total RNA Extraction Kit (Takara, Tokyo, Japan) according to the manufacturer’s instructions. RNA samples were then reverse transcribed into first-strand cDNA using the PrimeScript RT reagent kit from Takara. Real-time quantitative PCR was performed on an Applied Biosystems 7500 Real-Time PCR System (Life Technologies, Carlsbad, Calif). The amplification protocol was set as follows: 95°C denaturation for 30 seconds followed by 40 cycles of 5-second denaturation at 95°C, 30 s of annealing/extension, and data collection at 60°C. The primer sequences were listed in Table S3. Expression of GAPDH was served as a housekeeping gene for normalization. A ‘no template’ sample was used as a negative control. Relative gene expression was carried out with comparative 2−ΔΔCT method.

**Transfection**

HNEPC cells were transfected with small interfering RNA (siRNA) targeting CMPK2 (si-CMPK2) and siRNA targeting STING (si-STING) as previously described^12^. When reaching 60 to 80% confluence, HNEPC cells were transfected with si-CMPK2 (50 nM, Genepharma, Shanghai, China), si-STING (50 nM, RiBo biotechnology, Guangzhou, China) or their corresponding negative control siRNA (si-NC) using Lipofectamine RNAiMAX transfection agent (Thermo Fisher Scientific) in serum and antibiotic-free medium. After 6 hours, the medium was aspirated and replaced with medium containing serum for a further 24 or 48 hours. The sequences were listed in Table S4. In some experiments, cells were further stimulated with HDM (50 μg/mL) for 24 hours.

For plasmid transfection and mtDNA, when reached 60 to 80% confluence, HNEPC cells were transfected with plasmid encoding CMPK2 (RiBo biotechnology, Guangzhou, China) or empty plasmid by using Lipofectamine 3000 transfection agent (Thermo Fisher Scientific) according to the manufacturer’s instruction for 24 hours. In some experiments, cells were further treated with HDM (50 μg/mL) for 24 hours. After culture, cells were harvested and subjected to western blot analysis.

**Extraction and detection of mtDNA**

For the detection of mtDNA in the cytosol, HNEPC cells were plated at a density of 1 × 10^6^ cells/mL into 6 cm dishes. The cytoplasmic mtDNA of HNEPC cells was extracted using the Mitochondrial DNA extraction kit (ab65321, Abcam, Cambridge, MA, USA) as described in the manufacturer’s instructions^13^. Briefly, HNEPC cells were lysed in cytosol extraction buffer and placed in a dounce tissue grinder for 10 min. Next, the cells was homogenized and then centrifuged at 1200 g for 10 min at 4 °C to remove nuclei and intact cells. After that, the supernatant was collected and centrifuged at 10,000 g for 30 min at 4°C. The cytoplasmic mtDNA in supernate was extracted with DNA Extraction Kit (Takara, Tokyo, Japan). The level of mtDNA in cytosol was measured by qPCR assays, and Delta-Delta threshold cycle (2−ΔΔCT) was used for relative quantification. The primers used are listed in Table S5.

For mitochondrial DNA (mtDNA) copy number analysis, total DNA was extracted from HNEPC cells using DNA Extraction Kit (Takara, Tokyo, Japan). The amount of mitochondrial DNA relative to nuclear DNA was determined by quantitative real-time PCR using primers for ND1, ATP6, D-LOOP and 18S. mtDNA copy number was estimated by the comparative method using the 2–ΔΔCt.

**Animals**

CMPK2 KO mice were purchased from Cyagen Biosciences, and STING KO mice kindly provided by Professor Xiao Hui. All the animal experiments were approved by the Ethics Committee of Eye and ENT Hospital, Fudan University.

**In vivo experiment**

AR was induced in mice using HDM (Greer Laboratories, Lenoir, NC). Briefly, the mice were sensitized with an intraperitoneal injection of 100 μg together with Imject Alum (2 mg; Thermo Scientific, Waltham, MA, USA) in a total volume of 200 μl of PBS. One week after the last intraperitoneal injection, the mice were intranasally challenged with 50 μg of HDM solubilized in 20 μl of PBS on days 23 to 28. The control group was given saline. On day 29, the mice were anesthetized with avertin, and blood was collected from the orbital sinus. The mice were sacrificed by cervical dislocation, and samples of the septal and turbinate mucosa were collected for western blot analysis. For histological analysis, the mouse heads were fixed in 4% formalin for 24 h and decalcified in decalcification solution for three weeks. The experimental protocol is depicted in Figure 4A.

**References:**

1. Wise SK, Lin SY, Toskala E et al. International Consensus Statement on Allergy and Rhinology: Allergic Rhinitis. *Int Forum Allergy Rh*. 2018;8(2):108-352

2. Wise SK, Damask C, Roland LT et al. International consensus statement on allergy and rhinology: Allergic rhinitis - 2023. *Int Forum Allergy Rh*. 2023;13(4):293-859

3. Ritchie ME, Phipson B, Wu D et al. limma powers differential expression analyses for RNA-sequencing and microarray studies. *Nucleic Acids Res*. 2015;43(7):e47

4. Gu Z, Eils R, Schlesner M. Complex heatmaps reveal patterns and correlations in multidimensional genomic data. *Bioinformatics (Oxford, England)*. 2016;32(18):2847-2849

5. Szklarczyk D, Franceschini A, Wyder S et al. STRING v10: protein-protein interaction networks, integrated over the tree of life. *Nucleic Acids Res*. 2015;43(Database issue):D447-D452

6. Shannon P, Markiel A, Ozier O et al. Cytoscape: a software environment for integrated models of biomolecular interaction networks. *Genome Res*. 2003;13(11):2498-2504

7. Chin C, Chen S, Wu H et al. cytoHubba: identifying hub objects and sub-networks from complex interactome. *Bmc Syst Biol*. 2014;8 Suppl 4(Suppl 4):S11

8. Wu C, Orozco C, Boyer J et al. BioGPS: an extensible and customizable portal for querying and organizing gene annotation resources. *Genome Biol*. 2009;10(11):R130

9. Wang H, Zhu H, Zhu W et al. Bioinformatic Analysis Identifies Potential Key Genes in the Pathogenesis of Turner Syndrome. *Front Endocrinol*. 2020;11:104

10. Cao Y, Hu X, Zhou C et al. Increased IL-1α expression in chronic rhinosinusitis with nasal polyps. *European Archives of Oto-rhino-laryngology : Official Journal of the European Federation of Oto-Rhino-Laryngological Societies (EUFOS) : Affiliated With the German Society For Oto-Rhino-Laryngology - Head and Neck Surgery*. 2023;280(3):1209-1217

11. Xie Y, Li M, Chen K et al. Necroptosis Underlies Neutrophilic Inflammation Associated with the Chronic Rhinosinusitis with Nasal Polyps (CRSwNP). *Journal of Inflammation Research*. 2021;14:3969-3983

12. Zhou L, Zheng Y, Liao W et al. MUC1 deficiency promotes nasal epithelial barrier dysfunction in subjects with allergic rhinitis. *The Journal of Allergy and Clinical Immunology*. 2019;144(6)

13. Ning L, Wei W, Wenyang J, Rui X, Qing G. Cytosolic DNA-STING-NLRP3 axis is involved in murine acute lung injury induced by lipopolysaccharide. *Clinical and Translational Medicine*. 2020;10(7):e228
