# Supplementary material for: CMPK2 promotes NLRP3 inflammasome activation via mtDNA‐STING pathway in house dust mite‐induced allergic rhinitis
Source: Clin Transl Med. 2025 Jan 12;15(1):e70180. doi: 10.1002/ctm2.70180 (PMC11726638; doi:10.1002/ctm2.70180)

Uncropped gels for Westem Blots in Figure 1

F1-D

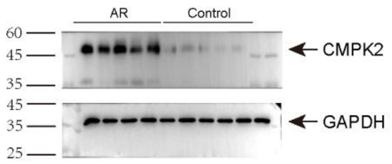

F1-F

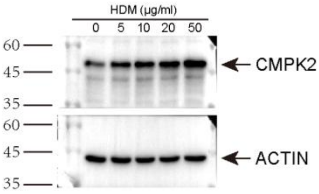

F1-G

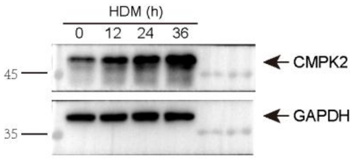

Uncropped gels for Westem Blots in Figure 3

F3-A

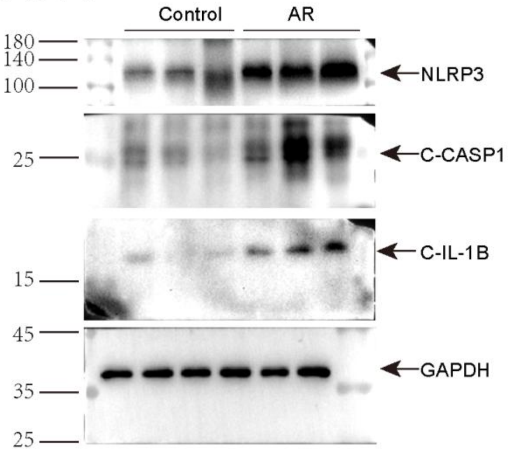

F3-B

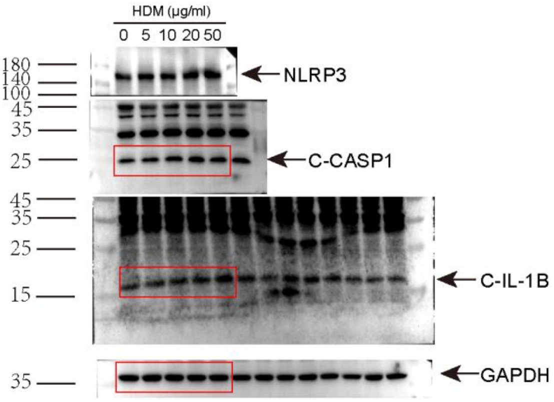

F3-E

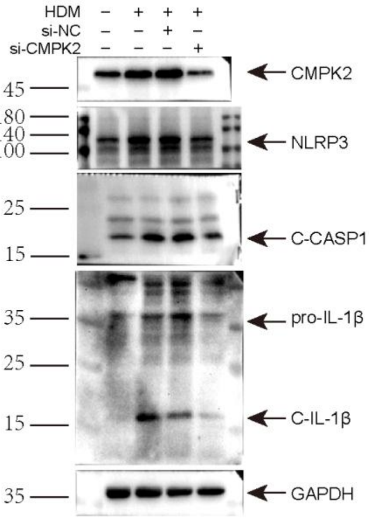

F3-F

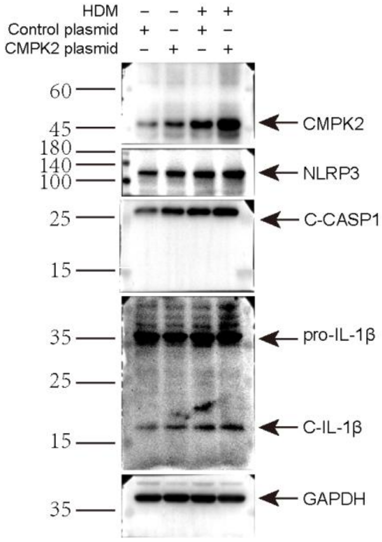

Uncropped gels for Westem Blots in Figure 4

F4-B

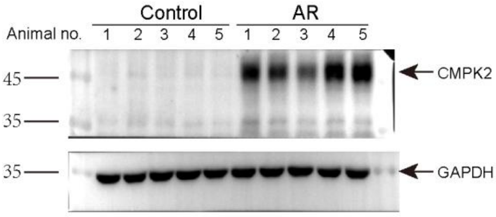

F4-D

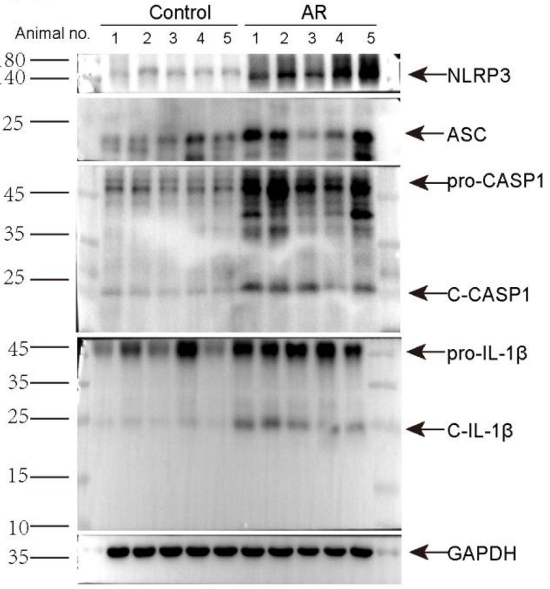

F4-I

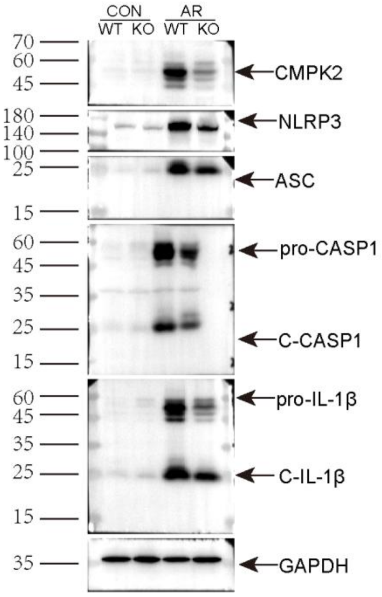

Uncropped gels for Westem Blots in Figure 5

F5-E

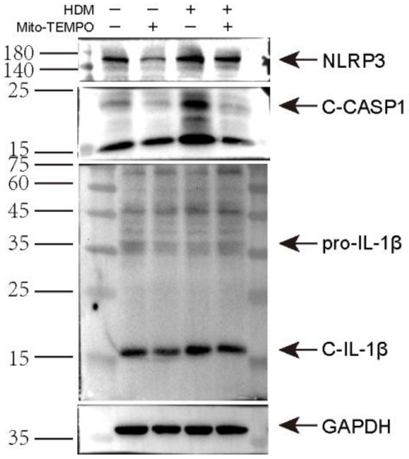

F5-K

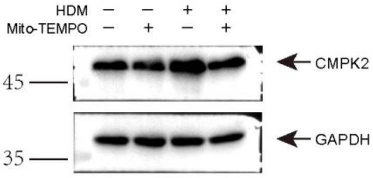

Uncropped gels for Westem Blots in Figure 6

F6-D

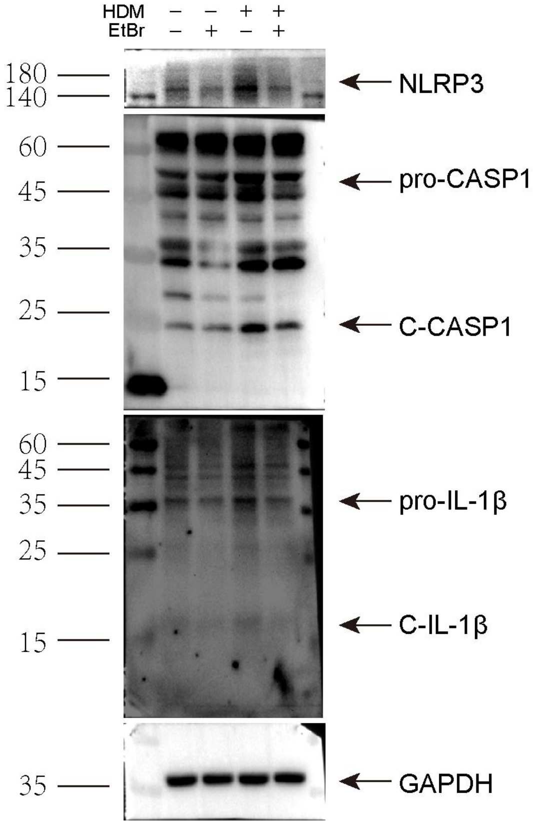

Uncropped gels for Westem Blots in Figure 7

F7-A

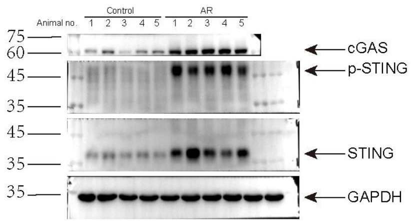

F7-B

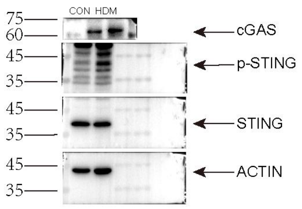

F7-E

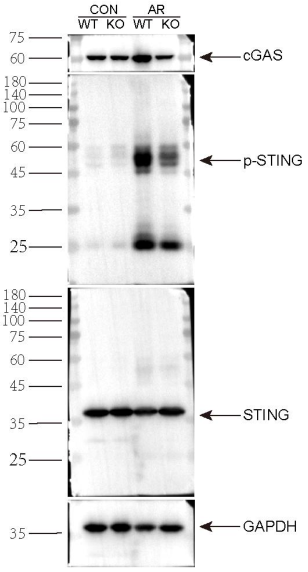

F7-F

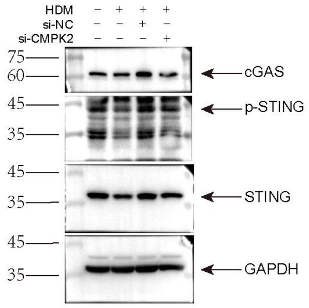

F7-G

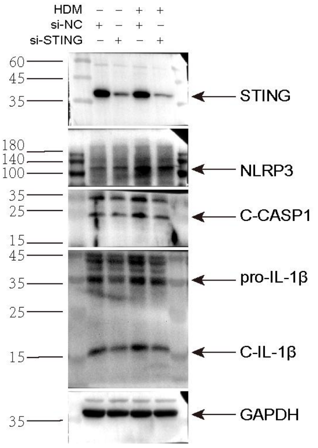

F7-H

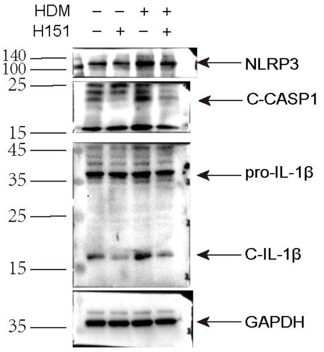

Uncropped gels for Westem Blots in Figure 8

F8-B

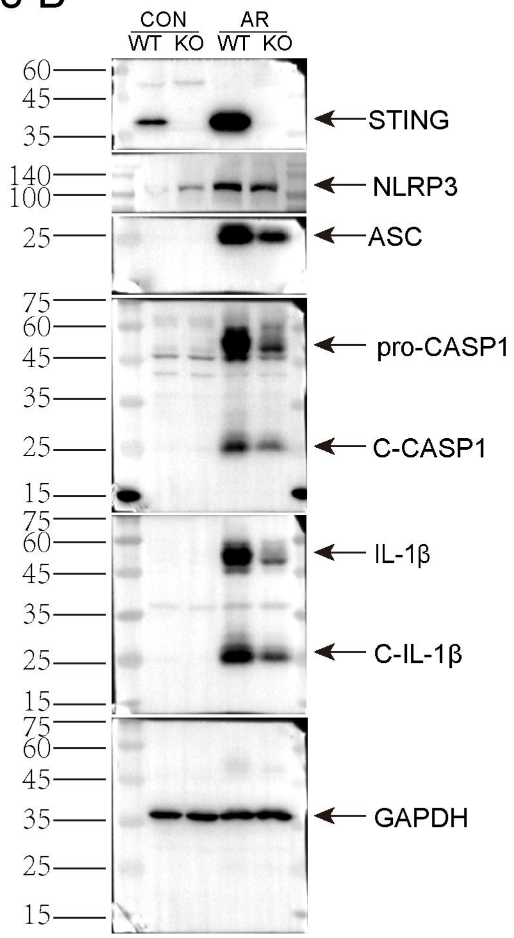

Uncropped gels for Westem Blots in Figure S1

FS1-G

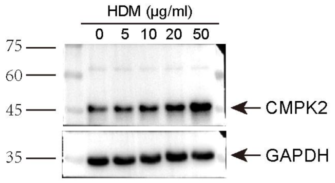

FS1-H

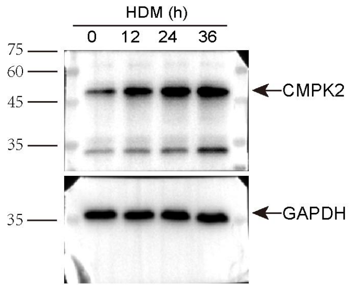

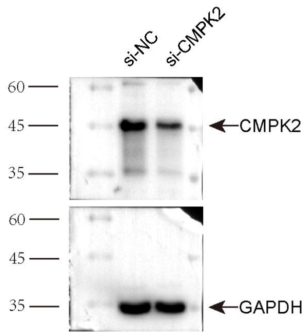

Uncropped gels for Westem Blots in Figure S2

FS2-A

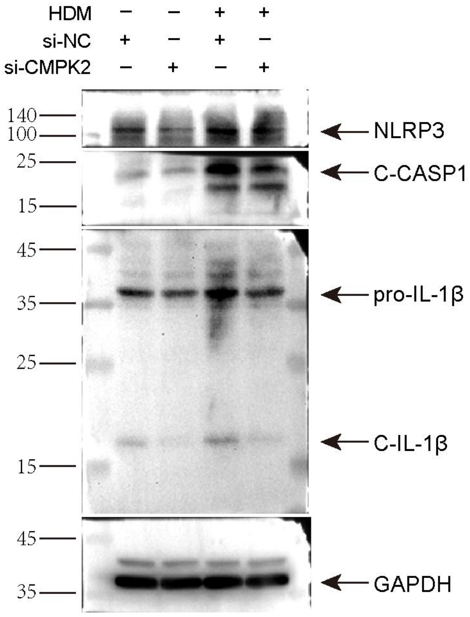

FS2-C

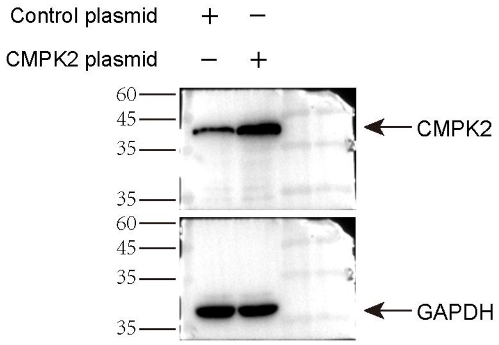

Uncropped gels for Westem Blots in Figure S3

FS3-A

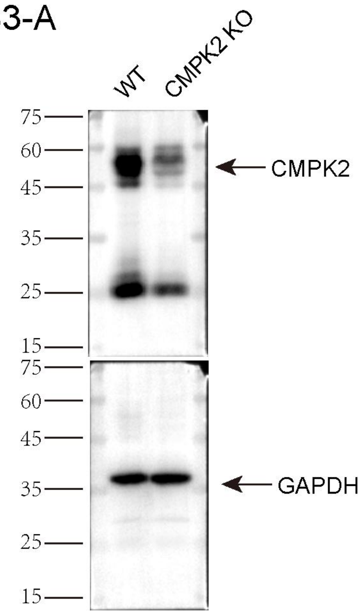

Uncropped gels for Westem Blots in Figure S7

FS7-C

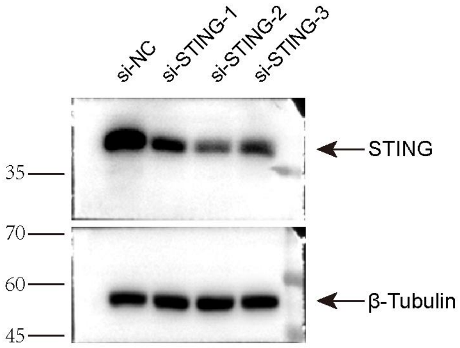

FS7-D

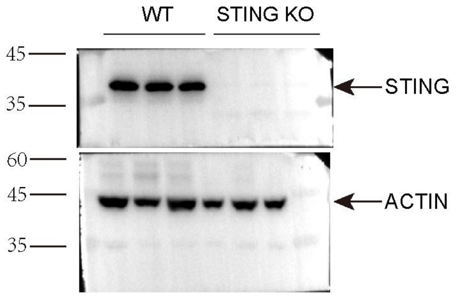

Uncropped gels for Westem Blots in Figure S8

FS8-A

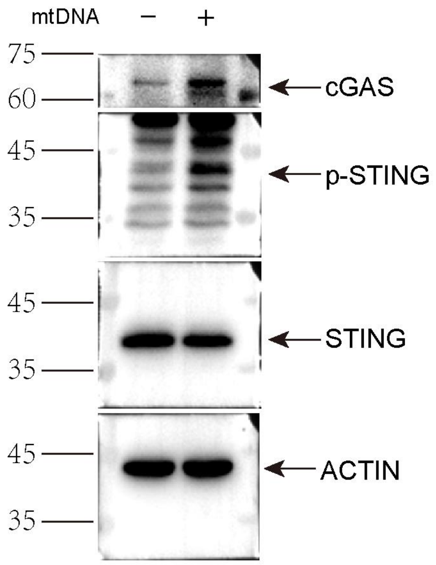

FS8-B

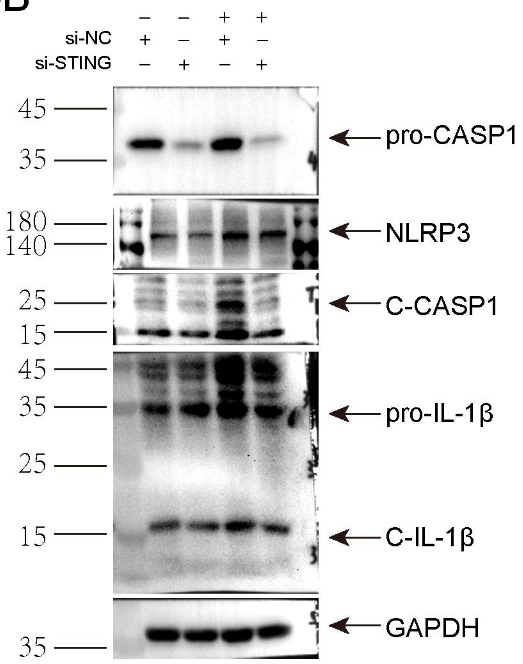

Uncropped gels for Westem Blots in Figure S9

FS9-A

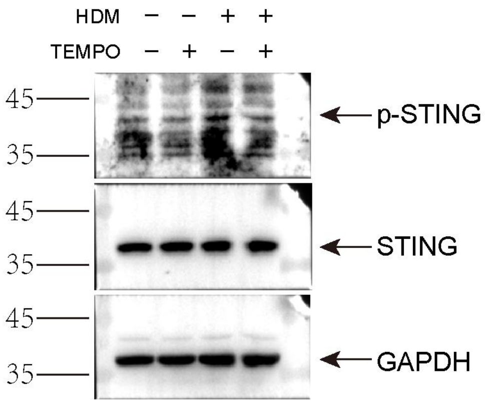

Supplement: Supplementary file 4 — Supporting Information [file CTM2-15-e70180-s003.pdf]
